# Technologies for investigating single-molecule chemical reactions

**DOI:** 10.1093/nsr/nwae236

**Published:** 2024-07-09

**Authors:** Chunyan Gao, Qinghua Gao, Cong Zhao, Yani Huo, Zhizhuo Zhang, Jinlong Yang, Chuancheng Jia, Xuefeng Guo

**Affiliations:** Center of Single-Molecule Sciences, Institute of Modern Optics, Frontiers Science Center for New Organic Matter, Tianjin Key Laboratory of Micro-Scale Optical Information Science and Technology, College of Electronic Information and Optical Engineering, Nankai University, Tianjin 300350, China; Center of Single-Molecule Sciences, Institute of Modern Optics, Frontiers Science Center for New Organic Matter, Tianjin Key Laboratory of Micro-Scale Optical Information Science and Technology, College of Electronic Information and Optical Engineering, Nankai University, Tianjin 300350, China; Center of Single-Molecule Sciences, Institute of Modern Optics, Frontiers Science Center for New Organic Matter, Tianjin Key Laboratory of Micro-Scale Optical Information Science and Technology, College of Electronic Information and Optical Engineering, Nankai University, Tianjin 300350, China; Center of Single-Molecule Sciences, Institute of Modern Optics, Frontiers Science Center for New Organic Matter, Tianjin Key Laboratory of Micro-Scale Optical Information Science and Technology, College of Electronic Information and Optical Engineering, Nankai University, Tianjin 300350, China; Center of Single-Molecule Sciences, Institute of Modern Optics, Frontiers Science Center for New Organic Matter, Tianjin Key Laboratory of Micro-Scale Optical Information Science and Technology, College of Electronic Information and Optical Engineering, Nankai University, Tianjin 300350, China; Hefei National Research Center for Physical Sciences at Microscale, University of Science and Technology of China, Hefei 230026, China; Center of Single-Molecule Sciences, Institute of Modern Optics, Frontiers Science Center for New Organic Matter, Tianjin Key Laboratory of Micro-Scale Optical Information Science and Technology, College of Electronic Information and Optical Engineering, Nankai University, Tianjin 300350, China; Center of Single-Molecule Sciences, Institute of Modern Optics, Frontiers Science Center for New Organic Matter, Tianjin Key Laboratory of Micro-Scale Optical Information Science and Technology, College of Electronic Information and Optical Engineering, Nankai University, Tianjin 300350, China; Beijing National Laboratory for Molecular Sciences, National Biomedical Imaging Center, College of Chemistry and Molecular Engineering, Peking University, Beijing 100871, China

**Keywords:** chemical reaction, scanning probe microscopy, single-molecule junction, single-molecule fluorescence, crossed molecular beam

## Abstract

Single molecules, the smallest independently stable units in the material world, serve as the fundamental building blocks of matter. Among different branches of single-molecule sciences, single-molecule chemical reactions, by revealing the behavior and properties of individual molecules at the molecular scale, are particularly attractive because they can advance the understanding of chemical reaction mechanisms and help to address key scientific problems in broad fields such as physics, chemistry, biology and materials science. This review provides a timely, comprehensive overview of single-molecule chemical reactions based on various technical platforms such as scanning probe microscopy, single-molecule junction, single-molecule nanostructure, single-molecule fluorescence detection and crossed molecular beam. We present multidimensional analyses of single-molecule chemical reactions, offering new perspectives for research in different areas, such as photocatalysis/electrocatalysis, organic reactions, surface reactions and biological reactions. Finally, we discuss the opportunities and challenges in this thriving field of single-molecule chemical reactions.

## Teaser

This revew summarizes a variety of single-molecule characterization techniques that have been developed for precise identification and detection of chemical reactions, advancing the understanding and applications of chemical reactions from a single-molecule perspective.

## INTRODUCTION

Molecules are the genetic building blocks of the material world, the key regulators of life processes and typical representatives of stable quantum systems, and their nature is of profound scientific significance. The essence of the way in which chemical reactions produce molecules lies in the breaking of old chemical bonds between atoms and the formation of new ones. Conventional chemical experiments are often based on statistical concepts through characterizing and describing the average properties and outcomes of the entire system, thereby obscuring the nuanced differences in individual properties. In contrast, single-molecule chemical reactions specifically refer to the microscopic behavior of individual molecules, focusing primarily on the reaction trajectories and reaction dynamics, as well as energy changes and potential energy surfaces during the reaction. These concepts play crucial roles in various research fields and applications.

Single-molecule chemical reactions focus studies on individual molecules. Under the influence of external factors like light, electricity, magnetism and force, even subtle changes in single molecules or atoms can be observed directly, allowing for real-time and *in-situ* monitoring of single-molecule chemical reaction dynamics, a task challenging to accomplish with ensemble averaging. By investigating the internal characteristics of individual molecules, such as energy level structures, excited states and vibrational modes, one can explore the essence of molecular interactions and energy transfer, revealing new behaviors, mechanisms and quantum processes that may be obscured by ensemble averaging effects [1–4]. These observations facilitate a more nuanced and comprehensive understanding of the intrinsic physical and chemical properties of molecules and chemical reaction mechanisms. Furthermore, this research allows for the exploration of unknown microscopic chemical phenomena that are inaccessible in ensemble experiments. Therefore, the development of single-molecule science and technology through the study of single-molecule chemical reactions holds immeasurable fundamental scientific value.

**Figure 1.**
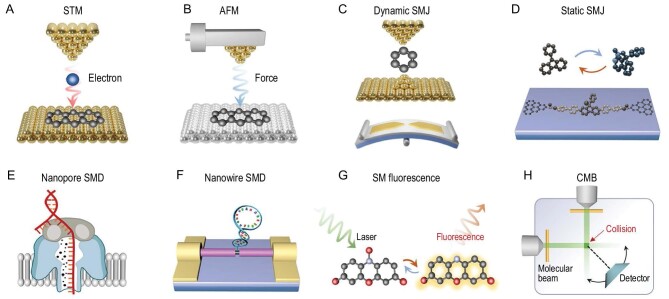
Single-molecule platforms for detecting chemical reactions. (A) Scanning tunneling microscope. (B) Atomic force microscope. (C) Dynamic single-molecule junction. (D) Static single-molecule junction. (E) Nanopore single-molecule detection. (F) Nanowire single-molecule detection. (G) Single-molecule fluorescence. (H) Crossed molecular beam.

The precise measurement of single-molecule chemical reactions stands as one of the ultimate challenges in current scientific explorations. Their development benefits from the advancement of ultrafine micro-nano fabrication and multidimensional extreme characterization techniques. Up to this point, there have primarily been five approaches, as illustrated in Fig. 1.

- Scanning probe microscopy (SPM), a high-resolution imaging technique, is widely used to study surfaces and nanostructures [5]. It allows for the observation and manipulation of individual atoms or molecules at the nanoscale with the advantage of atomic-level resolution, aiding in a deeper understanding of the properties and behaviors of the nanoworld. SPM techniques primarily include scanning tunneling microscopy (STM) based on the quantum tunneling effect, and atomic force microscopy (AFM) relying on various interaction forces (such as van der Waals and electrostatic attraction) [6–8].
- The single-molecule junction (SMJ) technique, which includes dynamic break junctions and static junctions, consists of a single conductive molecular bridge connecting two electrodes [9–11]. It allows for the control of the central molecule's chemical reactions through the application of external stimuli, such as light, electricity or force. The SMJ platform offers the advantage of studying reaction mechanisms without molecular labeling, which is attributed to its high time resolution in detecting reaction intermediates or even transition states through the use of electrical signals [12].
- Single-molecule detection (SMD) can be achieved based on single-molecule nanostructures, such as nanopores, nanowires and nanotubes. One-dimensional (1D) nanomaterials are materials with small sizes (usually no more than 100 nm) in a certain dimension, which can well match the size of a single molecule. Meanwhile, due to the large surface volumes and excellent electrical properties, the nanostructures have become an ideal platform for single-molecule measurements. The modifiable and label-free capabilities of 1D nanomaterials realize the stable and reliable connection of various molecules and provide endless opportunities for the study of single-molecule dynamics, molecular interactions and the revelation of molecular reaction mechanisms.
- Single-molecule fluorescence measurement is based on the fluorescence emission of target molecules or labeled dyes that are excited by a light source [13,14]. Then, according to the fluorescent spectra or imaging signals through the photon detectors, the information on conformational changes, dynamics, interactions and manipulation of single molecules can be tracked and decoded. Based on the high sensitivity of fluorescence detection and the extremely slight photon interference with target molecules, it has been widely used in studying the fluorescence lifetime and fluorescence resonance energy transfer of single molecules. Since this technology can reveal the correlation between the molecular structure and reaction mechanism, it is gradually emerging in real-time monitoring of chemical reaction pathways and will promote the rapid development of this field.
- The crossed molecular beam (CMB) technique is an experimental method used in the field of chemical physics to study the dynamics of molecular collisions. This technique focuses on the scattering behavior of molecules when two beams of molecules intersect at right angles. Subsequently, various methods are employed to detect the scattered molecules produced during collisions, including time-of-flight mass spectrometry, velocity mapping or other techniques that provide information about the products, their velocities and angular distributions. The CMB technique plays a crucial role in understanding fundamental processes such as molecular dynamics, reaction mechanisms and energy transfer in gas-phase chemical reactions.

This review primarily introduces the recent advancements, theoretical developments and current opportunities and challenges of single-molecule chemical reactions. The unique perspective provided by single-molecule chemical reactions offers new insights into various fields such as photochemistry, electrocatalysis, organic reactions, surface reactions and biochemistry. The exploration of interdisciplinary collaboration and research paradigms, the development of novel methodologies, the opening of new research domains, and the proposal of new theories all contribute to addressing critical frontiers in the fields of physics, chemistry, materials science and biology, with significant scientific and strategic implications.

## ON-SURFACE SINGLE-MOLECULE CHEMICAL REACTIONS

Precise manipulation and visualization of single-molecule chemical reactions has long been the goal of researchers, and are helpful for further investigation of chemical reactions and for revealing the mechanism of chemical reactions. With the advent of SPM techniques such as STM and AFM, it has become possible to image the chemical, electronic and electrostatic properties of individual molecules at the sub-molecular level [15,16]. This technology enables the tracking of complex surface chemical reactions, the study of underlying principles and the synthesis of new functional molecules. On-surface chemistry provides a new platform for studying molecular systems that were previously difficult to investigate using traditional chemical methods. It allows the characterization of the structure, chemistry and physical properties of relevant materials at the single-chemical-bond scale.

### Theoretical basis of single-molecule chemical reactions

The theory of single-molecule chemical reactions is crucial in exploring reaction mechanisms. This theory outlines a series of steps in which chemical reactions occur, encompassing reactant interactions, and intermediate formation and transformation, as well as the generation of final products. Over the past century, the theory has rapidly advanced alongside the consistent evolution of experimental techniques and computational methods, offering a vital instrument for gaining profound insights into the essence and intricacies of chemical reactions.

The behavior of single molecules during chemical reactions can be described by the theory of single-molecule reaction dynamics, which incorporates both classical theory and quantum-based theory [17,18]. The primary focus of the classical case is on the evolution and motion of a molecule along the potential energy surface, and how the evolution impacts the mechanism and rate of reactions [17]. According to this theory, the state of a molecule, including its position, velocity and acceleration, is governed by the laws of motion in classical mechanics. The potential energy surface during the reaction process encompasses all possible states, such as multiple local minima and saddle points on the potential energy surface, which typically correspond to reactants, intermediates and transition states. Classical theory assumes the deterministic motion of a molecule on the potential energy surface, implying that, given initial state conditions (such as position and velocity), the trajectory is unique. By solving the equations of motion in classical mechanics, one can determine the trajectory of a molecule on the potential energy surface, enabling the analysis of mechanisms and reaction rates. Moreover, the reaction rate can be influenced by the energy barriers, and the classical theory can calculate the residence time of a molecule on the potential energy surface and the possibility of overcoming an energy barrier to derive the reaction rate constant. It is noteworthy that classical theory has certain limitations. For instance, it cannot encompass the influence of quantum effects, such as tunneling, on reaction rates. Furthermore, it cannot precisely depict microscopic behaviors, such as vibrations and rotations of molecules on the potential energy surface. Consequently, in practical applications, classical theory often requires integration with other methods and theories to offer a more precise description and prediction of the dynamic behavior of single-molecule chemical reactions.

The quantum-based theory of single-molecule reaction dynamics leverages quantum mechanics principles and techniques to characterize the behavior of single molecules throughout chemical reactions [18]. Unlike the classical case, the quantum-mechanical approach affords a more meticulous portrayal of electronic configurations and actions of a molecule during reactions, thus enabling more precise forecasts of reaction mechanisms and rates. In quantum-based theory, the state of a molecule is represented by the wave function, encapsulating all conceivable states and their corresponding probabilities. By solving the Schrödinger equation for the molecule, one can ascertain its energy and wave function, enabling the exploration of the electronic structure of the molecule and the identification of reaction barriers and paths. Furthermore, critical metrics like vibration frequencies and reaction activation energies can be computed to unravel the reaction mechanism and rate-limiting factors, offering a crucial theoretical backdrop for the design and refinement of chemical reactions.

### Development and principles of scanning probe microscopy

SPM is a technique that enables the imaging and manipulation of surfaces at the molecular and even atomic scale, ushering in a paradigm shift in our understanding and perception of matters at the nanoscale and molecular level. Typically, it entails a sharp probe scanning across the sample surface and integrating nanoscale sensors at the probe tip or coupling various electromagnetic waves with the probe–sample junction to collect information about the sample's surface properties [19]. This provides the opportunity to gain insight into electronic, vibrational, optical, magnetic, biochemical and mechanical characteristics of the molecular structure. Currently, a variety of SPMs have been widely applied in research. STM, a groundbreaking invention by Binnig and Rohrer in 1982, has ushered in a new era of surface science exploration [20]. For their remarkable contribution, they were awarded the Nobel Prize in Physics in 1986. That very year, AFM emerged as a result of the innovative collaboration between Binnig and Quate [7]. They ingeniously merged the fundamental principles of STM with the stylus profilometer, enabling the measurement of ultra-tiny forces on particles as small as single atoms. In 1990, STM was first used to position individual atoms with atomic precision at low temperatures (4 K), laying the foundation for atomic-level structural assembly [21]. After that, Sugimoto *et al*. performed the atom manipulation of Si atoms at room temperature [22]. With ongoing in-depth research, STM can also be employed to manipulate chemical reactions. Hla *et al*. induced all steps of a chemical reaction with STM [23]. In 2009, atomic resolution imaging of molecules was achieved through the manipulation of the tip of an atomic force microscope with functionalized tips, providing a more detailed understanding of chemical reactions and catalysis [24]. In general, SPM can be mainly divided into two categories: AFM and STM.

AFM relies on extremely weak interaction forces (van der Waals forces or electrostatic attraction) between the sample surface and a tiny-force-sensitive element to study the topological structure, morphology and physical properties of molecules on the substrate surface. It is equipped with a highly sensitive microcantilever, one end of which is connected to a piezoelectric displacement actuator, while the other end contains a probe tip that interacts with the sample. As the tip scans across the surface, the forces between the tip and the sample cause deflections of the cantilevers. These deflections are detected using a laser beam reflected from the cantilevers and are used to reconstruct the surface topography. AFM can operate in different modes, including contact mode (the tip maintains constant contact with the sample) and non-contact mode (the tip oscillates close to the surface). To achieve atomic-level resolution in imaging and functional applications of the properties, targeted modifications need to be made to the probe tip. For example, by adsorbing gases such as N_2_, O_2_, Cl_2_ or CO on the tip, the interaction forces between the surface and the tip can be adjusted, thus allowing for highly precise atomic-level resolution using a *q*Plus, an extremely sensitive force-sensing system. Alternatively, by attaching specific molecules to the tip, specific chemical interactions or recognition can be achieved, making it useful for applications such as molecular recognition and the measurement of biomolecules.

STM relies on the quantum tunneling phenomenon. By applying a bias voltage between an atomic-level metal tip and a conductive molecule, a potential drop is created. The moving probe can interact with the material's surface, and the tunneling current between the surface and the tip reflects the electronic transitions at the molecular level between the surface atoms and the probe. This enables the study of molecular surface electronic structures, molecular orbitals and local electron density states. By measuring the tunneling current, STM can provide real-time observation of the individual atoms’ arrangement on a molecular surface and properties related to the surface electronic behavior. It offers high-resolution imaging and detailed molecular information, providing powerful technical support for fundamental studies of basic molecular properties.

### Detection of on-surface inorganic small-molecule chemical reactions

In many typical small molecules, water plays a crucial role in various physical, chemical and biological processes, serving as the foundation for numerous reactions. SPM is also one of the important techniques for studying on-surface small-molecule chemical reactions, delving into the structure, electronic property, and behavior of molecules. Representative studies of surface photocatalytic reactions have been widely conducted since Fujishima and Honda first demonstrated the photocatalytic splitting of water (H_2_O) on a TiO_2_ electrode in 1972 [25], especially in the investigation of small-molecule chemical reaction mechanisms in titanium dioxide photocatalysis processes.

**Figure 2.**
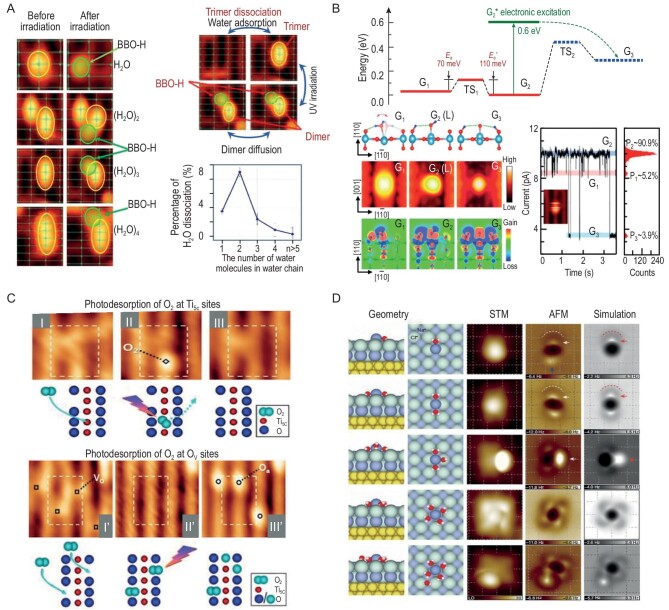
On-surface inorganic small molecule chemical reactions. (A) STM images and percentage of surface H_2_O molecules photoinduced dissociation of different species. Adapted with permission from ref. [26]. Copyright 2016 American Chemical Society. (B) DFT calculations and stationary geometries of molecular and dissociative D_2_O with *I–t* curves. Adapted with permission from ref. [27]. Copyright 2019 American Chemical Society. (C) STM images show photodesorption of O_2_ from the Ti_5c_ site and the O vacancies site. Adapted with permission from ref. [28]. Copyright 2011 American Chemical Society. (D) Geometries and high-resolution STM/AFM images of Na^+^ hydrates. Adapted with permission from ref. [29]. Copyright 2018 Macmillan Publishers Limited.

For hydrolysis reactions, Yang *et al.* conducted research on the role of hydrogen bonds in photoinduced water dissociation on the R-TiO_2_(110) surface using STM and programmed temperature desorption techniques (Fig. 2A) [26]. After adsorbing ∼0.08 mL H_2_O at ∼80 K, four distinct types of images were observed on the R-TiO_2_(110) surface. These images correspond to H_2_O monomer, dimer, trimer and tetramer on the Ti_5c_ sites. Subsequently, after the surface was irradiated with 266 nm light for 60 min, it became evident that some of the monomers adsorbed on the Ti_5c_ sites dissociated. During this process, a single hydrogen atom was transferred to the adjacent bridge-bonded oxygen (BBO) site, leading to the disappearance of the OH radical at the Ti_5c_ site. It is worth noting that the single hydrogen bond between two H_2_O molecules significantly enhances the reactivity of the H_2_O acceptor molecule in the dimer on the R-TiO_2_(110) surface, while the 1D hydrogen bonding between surface H_2_O molecules strongly inhibits the H_2_O dissociation reaction. The strong hydrogen bonding network between water molecules is a critical factor in water splitting.

Wang *et al.* precisely measured the water dehydrogenation process at the stoichiometric Ti_5c_ sites on the TiO_2_(110) surface (Fig. 2B) [27]. The hydrogen bond dynamics of H_2_O and D_2_O indicate that vibration and electronic excitation dominate the sequential transfer of two H (D) atoms from H_2_O (D_2_O) molecules to adjacent surface oxygen sites, highlighting the active involvement of the oxide surface in the dehydrogenation process. The research findings suggest that at the stoichiometric Ti_5c_ sites, the energy stability of a single H_2_O molecule is lower than that of the dissociative form. Both experimental and theoretical results demonstrate that the potential well of the dissociative form is expected to be as small as ∼70–120 meV. At 0.7 V, a typical current-time (*I−t*) curve exhibits three distinct conductance states, which can be correlated with their respective steady-state heights, with G_2_ > G_1_ > G_3_. In addition, the calculated charge difference maps depict the charge redistribution for three different geometries, indicating that the surface O_b_ atom serves as a hydrogen bond acceptor for D₂O molecules (G_1_), while it acts as a hydrogen bond donor for dissociated D₂O (G_2_ and G_3_). Hence, the jumps in the *I−t* curve and the change in the contrast of STM images can be attributed to changes in the direction of hydrogen bonds during the transfer of H or D atoms. These results suggest that interface hydrogen bonds can effectively assist the transfer and exchange of hydrogen atoms on the surface. The revealed quantitative hydrogen bond dynamics provide a new atomic mechanism for the interaction between water and metal oxides.

In addition, the photocatalytic reactions of single O_2_ molecules on the reduced TiO_2_(110) surface were imaged and monitored for the first time using STM, as shown in Fig. 2C [28]. To properly describe two complementary redox channels (O_2_ desorption and dissociation), additional active sites need to be considered. Density functional theory (DFT) calculations indicate that the coordination and charge state of O_2_ chemisorbed at specific sites largely determine the particular reaction pathways. The two O_2_-involved reactions follow significantly different kinetic patterns with desorption being much faster than dissociation, and the latter being inhibited above a certain O_2_ coverage. These results are applicable when O_2_ coverages reach O vacancy concentration and should be regarded as the first step in studying O_2_ photochemistry on TiO_2_(110). It is expected that higher oxygen coverages will also involve similar, site-specific photoresponsive channels.

Water molecules are commonly presented in the form of hydrated ions in chemical and biological systems rather than existing as independent entities, making it crucial to understand the structure of hydrated ions. In recent studies, Jiang *et al.* not only achieved atomic-level resolution images of hydrated ions, but also employed a charged tip as an electrode to control the directed transport of individual hydrated ions on the surface of NaCl (Fig. 2D) [29]. At the same time, an interesting magic number effect has been discovered: sodium ion hydrates containing a specific number of water molecules exhibit an exceptionally high diffusion ability, with their mobility being several orders of magnitude higher than that of other hydrates, even surpassing the mobility of bulk-phase ions. Combining first-principle calculations and classical molecular dynamics simulations, it has been shown that this magic number effect arises from the symmetric matching between hydrated ions and the surface lattice. This work establishes a direct connection between the microstructure of hydrated ions and their transport properties, challenging conventional concepts of ion transport in confined systems. Hydronium ions are an important form of hydrate, typically found in acidic solutions, represented as H_3_O^+^. This form of hydrate is crucial for understanding acid-base reactions and the ion chemistry and ion transport processes in aqueous solutions. The microscopic structure of hydrated hydrogen ions has been observed using the next-generation *q*Plus-type force sensor, which greatly improved the quality factor and minimum detection force through its optimized design. Studying the intrinsic properties of inorganic small molecules using SPM technologies is beneficial for a deeper molecular-scale understanding of chemical reaction processes, revealing the roles and behaviors of molecules in chemical reactions, and aiding in further comprehension of processes such as dissolution, catalysis and chemical reaction dynamics.

### Detection of on-surface organic small-molecule chemical reactions

For organic small molecules, achieving precise characterization of molecular structures at the interface has long been challenging in the field of chemistry. Contemporary advanced tip-based microscopy techniques now permit the resolution of molecular heterogeneity at the single-molecule level by analyzing distinctive electronic, geometric and vibrational properties.

**Figure 3.**
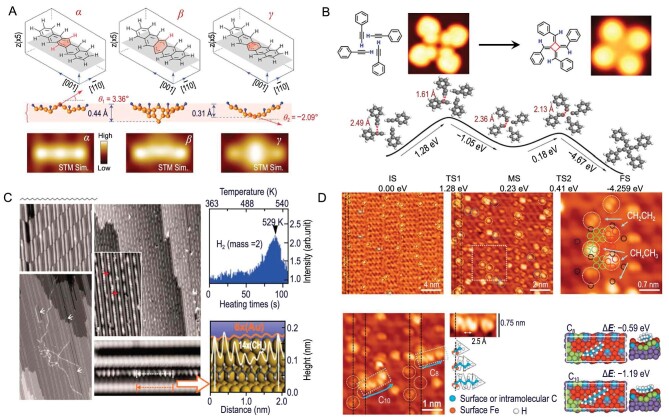
On-surface organic small-molecule chemical reactions. (A) Optimized structures and simulated images of three pentacene species. Adapted with permission from ref. [30]. Copyright 2021 American Association for the Advancement of Science. (B) Cyclotetramerization reaction of self-assembled alkyne groups with DFT calculations and corresponding energy profiles. Adapted with permission from ref. [31]. Copyright 2018 Nature Publishing Group. (C) STM images for dehydrogenative polymerization of *n*-dotriacontane on Au(110) with height profiles and H_2_ signal. Adapted with permission from ref. [32]. Copyright 2011 American Association for the Advancement of Science. (D) STM images of the carburized Fe(110) exposed to ethylene at room temperature. Adapted with permission from ref. [33]. Copyright 2022 American Association for the Advancement of Science.

Wang *et al.* utilized SPM in conjunction with tip-enhanced Raman scattering (TERS) techniques to fully map individual vibrational modes and construct the chemical structure of individual molecules intuitively, as shown in Fig. 3A [30]. This method facilitated precise measurements of single molecules influenced by external fields, including electrical, mechanical and photonic forces, thus achieving detailed characterization with enhanced specificity at the level of individual chemical bonds. Specifically, using the Ag(110) surface and pentaphene (C_22_H_14_) as a model system (denoted as *α*), the continuous application of a 2.0 V voltage pulse on the molecule induced the formation of two distinct species, *β* and *γ*, with different shapes. Species *β* exhibited a slightly distorted dumbbell shape, while species *γ* had a spindle-like shape. The SPM-TERS combined strategy provides a comprehensive means to explore the structure and chemical heterogeneity of surface species. This experimental approach holds broad applicability in surface chemistry and catalysis research, particularly at the single-bond limit.

In addition to achieving the precise characterization of interfacial molecular structures, SPM has also found extensive application in the field of organic cyclic small molecules. For instance, tetra-methylenecyclobutane ([4]radialene) and its derivatives have garnered widespread theoretical and synthetic interest due to their unique cross-conjugated eight-center eight-π electron systems. The core region of this molecule not only requires a four-membered ring composed of four carbon-carbon single bonds, but also mandates that the carbon atoms forming the four-membered ring are directly connected to carbon-carbon double bonds outside the ring. Chi *et al.* reported a method involving low-temperature adsorption on the Cu(100) surface (20 K) followed by slow annealing. Through [1 + 1 + 1 + 1] direct cyclization reaction, they selectively synthesized tetraquinene. Simultaneously, high-resolution SPM was employed for *in-situ* characterization of the reaction, and the reaction mechanism was elucidated through a combination of reaction kinetics calculations (Fig. 3B) [31]. The method of low-temperature adsorption (20 K) followed by slow annealing suppresses the migration of molecules on the surface and induces the formation of an intermediate structure containing four phenylethyne supramolecules. In the process of generating the intermediate structure of the supramolecule, the Cu(100) fourfold symmetric lattice serves as an excellent template. Furthermore, the catalysis by surface copper atoms significantly reduces the energy barrier for the [1 + 1 + 1 + 1] direct cyclization reaction. Calculations indicate that the barrier for the cyclization reaction is 0.32 eV, only 0.04 eV higher than the barrier for the surface migration of phenylethyne molecules. Once the intermediate structure of the tetramer supramolecule is formed, the cyclization reaction immediately occurs. This study emphasizes the crucial role of the novel approach of suppressing the migration capability of phenylacetylene on the copper metal surface. The migration of molecules on the surface plays a vital role in product formation, and one can control the kinetic factors of relevant reactions to select the process of surface reactions.

For classical linear alkanes in organic chemistry, there are few schemes that predictably couple saturated hydrocarbons, compared to many methods for selectively coupling olefin polymers. Chi *et al.* discovered a high-selectivity carbon-hydrogen (C−H) activation and subsequent dehydrogenative C−C coupling reactions of long linear alkanes (>C_20_) on an anisotropic Au(110) surface. This method involves appropriate reconstruction through molecular adsorption and subsequent mild annealing, resulting in nanosized channels (width of 1.22 nm) (Fig. 3C) [32]. Due to the orientation constraints of reactant molecules in these 1D channels, the reaction occurred only at specific positions on the chain (terminal CH_3_ or the second CH_2_ group) at intermediate temperatures (420–470 K). The reaction exhibited selective aliphatic C−H activation rather than aromatic C−H activation.

Furthermore, it is important to explore the mechanisms of industrial catalytic systems and establish controllable and well-ordered model systems in the study of single-molecule reactions. Recently, Wu *et al.* made progress in visualizing the ethylene polymerization process on a carburized iron surface (Fig. 3D) [33]. Catalyzing the production of polyethylene through the polymerization of ethylene is one of the most common processes in the chemical industry. The popular Cossee-Arlman mechanism assumes the direct insertion of ethylene into the metal-carbon bond during chain growth, a hypothesis that has been awaiting confirmation through microscale and temporal experiments. This work achieved *in-situ* visualization of the ethylene polymerization process on a carburized iron single-crystal surface using STM. They observed specific triangular iron sites at the boundary between two carbide domains where ethylene polymerization took place. In the absence of an activator, the surface-anchored vinyl (−CHCH_3_) intermediates acted as chain initiators (autoinitiation), which subsequently grew through ethylene insertion. This finding provides direct experimental evidence at the molecular level for the ethylene polymerization pathway. Therefore, taking advantage of the precise characterization of SPM, the occurrence of on-surface organic small-molecule chemical reactions can be detected, and the reaction dynamic process can be further revealed.

### Accurate manipulation of on-surface organic small-molecule chemical reactions

**Figure 4.**
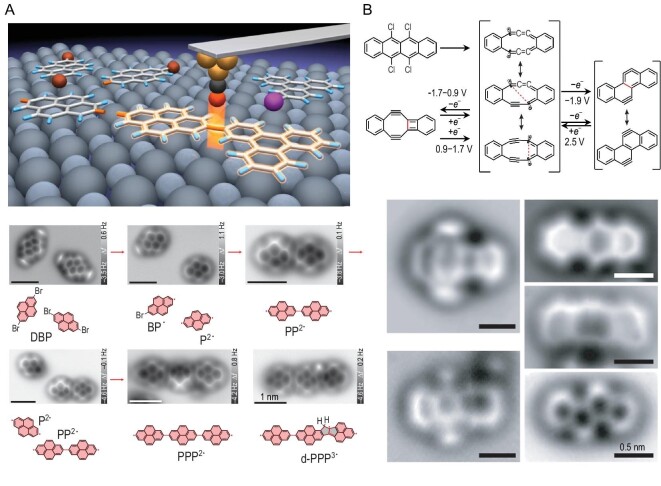
Atomically accurate tip-induced synthesis. (A) Tip-induced deiodination and intermolecular homo-coupling of 2-iodotriphenylene. A few representative AFM images illustrate the main steps for the manipulation, as follows: two isolated DBP molecules; a BP^•^ monoradical and a P^2•^ diradical; a PP^2•^ homo-dimer formed via homo-coupling of two pyrene diradicals; a P^2•^ diradical and a PP^2•^ homo-dimer; a PPP^2•^ homo-trimer; a defective pyrene trimer d-PPP^3•^ with two fused pentagons generated by skeletal rearrangement. Scale bars: 1 nm for all AFM images. Adapted with permission from ref. [34]. Copyright 2021 Springer Nature Limited. (B) Reaction scheme and AFM images of 5,6,11,12-tetrachlorotetracene. Adapted with permission from ref. [35]. Copyright 2022 American Association for the Advancement of Science.

Precise control of the reaction pathways of individual molecules is a central goal in the field of chemistry. The SPM technique allows for the manipulation of single molecules by controlling the probe tip and achieving precise control over single-molecule chemical reactions through techniques such as tip-induced charge injection or photoillumination. For instance, Zhong *et al.* have used SPM to control molecules one by one to construct covalent organic nanoarchitectures, as shown in Fig. 4A [34]. Firstly, a bilayer NaCl film was grown on the Cu(111) surface as a substrate, followed by the deposition of various halogenated aromatic molecules onto the NaCl film at a low temperature (5 K). Through scanning probe manipulation, an electrical excitation method was employed based on the inelastic tunneling of electrons to control the activation, movement and coupling reactions of the molecules. Specifically, by selectively connecting two different molecules, control of the chemical site and regional selectivity, as well as two-dimensional (2D) stereo selectivity, was achieved. Selective bonds were formed based on the specificity of the triphenylene and pyrene structures, demonstrating the controllability and design freedom of single-molecule chemical reactions. The selective covalent assembly of multiple organic structural units demonstrated that diverse molecular structural elements can be connected in a controlled manner through traditional thermal coupling methods. This paves the way for synthesizing elusive covalent nanostructures, studying structural modifications and revealing pathways for intermolecular reactions. It also provides a direction for tip-induced, atomically precise molecular assembly.

**Figure 5.**
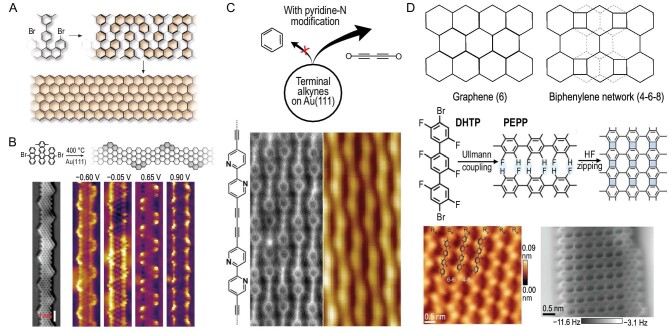
On-surface organic nanostructure synthesis. (A) Zigzag graphene nanoribbons (GNRs). Adapted with permission from ref. [36]. Copyright 2016 Springer Nature Limited. (B) Armchair GNRs. Adapted with permission from ref. [37]. Copyright 2018 Springer Nature Limited. (C) Graphdiyne (GDY) nanowires. Adapted with permission from ref. [38]. Copyright 2023 American Chemical Society. (D) Biphenylene network. Adapted with permission from ref. [39]. Copyright 2021 American Association for the Advancement of Science.

**Figure 6.**
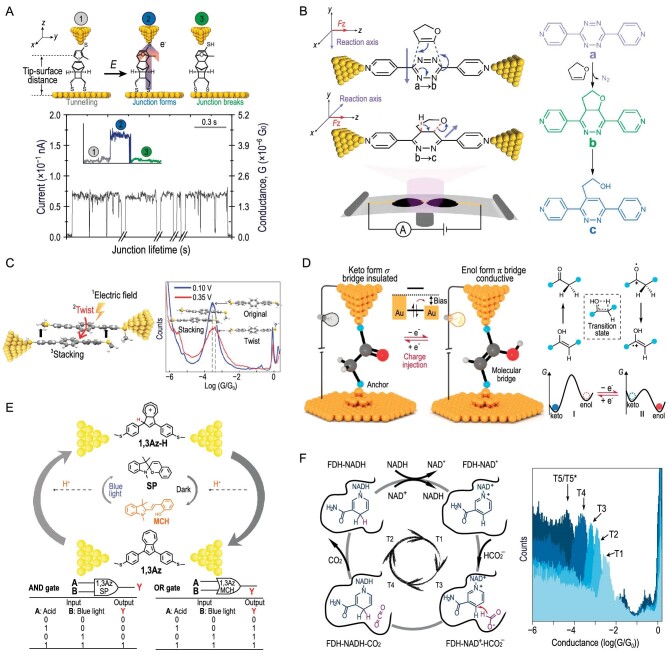
Single-molecule chemical reactions in dynamic single-molecule break junctions. (A and B) Electrostatic catalysis of a Diels-Alder reaction based on STM-BJ (A) and MCBJ (B). Adapted with permission from ref. [46,47]. Copyright 2016 Macmillan Publishers Limited and 2019 American Association for the Advancement of Science. (C) Assembly schematic and theoretical simulation diagram of electric-field-induced terphenyl structure based on STM-BJ. Adapted with permission from ref. [50]. Copyright 2020 American Chemical Society. (D) Schematic diagram of nanoscale device based on single-molecule keto-enol equilibrium, including the scheme of keto-enol tautomerization reaction and schematic diagrams of PESs I and PESs II under different charged states. Adapted with permission from ref. [51]. Copyright 2023 Nature Publishing Group. (E) Illustration of intermolecular proton transfer in light-driven non-photoresponsive 1,3-azulene derivative 1,3Az, along with the truth table for molecular AND gate and molecular OR gate. Adapted with permission from ref. [52]. Copyright 2019 Wiley-VCH Verlag GmbH & Co. KGaA, Weinheim. (F) Schematic and conductance histograms for the catalytic cycle of FDH based on STM-BJ. Adapted with permission from ref. [56]. Copyright 2023 Springer Nature Limited.

For a long time, the manipulation of individual molecular chemical bonds by external electric fields and charge states has been a challenging field. Gross *et al*. demonstrated the precise manipulation of the breaking and forming of individual chemical bonds using voltage pulses at the tip of STM, achieving reversible and controlled transformations between three isomers [35]. As shown in Fig. 4B, 5,6,11,12-tetrachloro-terphenyl was firstly sublimated onto a Cu(111) substrate, partially covered with one to three monolayers of sodium chloride. At a low temperature of 5 K, when the voltage at the tip of the STM is around +3.5 V and the tunneling current is in the order of 1 pA, two Cl atoms on 5,6,11,12-tetrachloro-terphenyl dissociate. The molecule can also be moved several nanometers on the surface. When voltage pulses are applied between 4 and 4.5 V, the remaining chlorine atoms also dissociate, forming different isomers with the chemical formula C_18_H_8_. At this point, applying different bias voltages allows for the transformation between isomer structures. STM provides a method for achieving reaction selectivity by controlling the input voltage, enabling the controlled transformation of unstable molecules. The tip-induced electrochemical studies of oxidation-reduction reactions revealed the rules governing redox processes between the isomers. A deep understanding of these rules contributes to a better comprehension of important redox reactions in organic synthesis and nature. For the development of future artificial molecular machines, the controllable, reversible and selective switching of reactions holds significant importance.

### Detection of on-surface organic nanostructure synthesis reactions

SPM can provide an effective technique for detecting surface chemical syntheses, enabling the study of molecular systems that were previously challenging to investigate using traditional chemical methods. Based on a ‘bottom-up’ approach to surface synthesis, it combines various surface techniques such as non-contact AFM, STM and scanning tunneling spectroscopy, to monitor and characterize the basic structure, chemical properties and physical properties of molecules.

In recent years, the development of surface chemistry synthesis methods has enabled the controlled growth of organic nanomaterials. The electronic properties of graphene-based nanostructures are directly correlated with their structural boundary conditions. Zigzag edges hold the potential to host spin-polarized electronic edge states, making them promising candidates as key components of graphene-based spintronics. Moreover, armchair graphene nanoribbons have been found to induce significant electronic bandgap openings. Different precursor monomers allow for precise control over the width, shape and doping of the nanostructures. The precursor monomers (2,12-dibromo-14-(3′,5′-dimethyl-[1,1′-biphenyl]-4-yl)-dibenzo[a, j]anthracene) undergo a dehalogenation activation on the Au(111) single-crystal surface at 475 K, followed by polymerization through radical addition. Subsequently, a further annealing cyclodehydrogenation reaction takes place at 625 K, resulting in the formation of the final Zigzag graphene nanoribbon (Fig. 5A) [36]. The 6,11-bis(10-bromoanthracen-9-yl)-1,4-dimethyltetracene, as a precursor monomer, undergoes thermal precursor activated-dehalogenation, polymerization and cyclodehydrogenation on the Au(111) surface, and the armchair graphene nanoribbon with quantum state topological properties has been successfully synthesized, as shown in Fig. 5B [37]. This armchair graphene nanoribbon with topological electronic bands could be described by a 1D tight-binding Hamiltonian, creating quasi-1D electronic quantum phases. Moreover, there is the prospect of tuning the bandwidth of the topological electronic bands to be close to the energy scale of induced spin-orbit coupling or superconductivity, potentially realizing Kitaev-like Hamiltonians and Majorana end states.

In addition, on-surface chemistry synthesis has a natural advantage in enhancing chemical selectivity. Based on SPM, the acetylenic homocoupling reaction of polarized terminal alkynes on Au(111) could effectively avoid the formation of enyne products or cyclotrimerization products. Specifically, pyridine nitrogen modification is used to differentiate the C−C coupling motifs, inhibiting cyclotrimerization and promoting linear coupling to produce well-arranged N-doped graphdiyne nanowires (Fig. 5C) [38]. The repetitive motifs along one dimension in these carbon nanostructures have sparked research into the properties of planar carbon allotropes. While planar *sp*^2^-hybridized carbon allotropes have garnered significant attention due to their unique mechanical, electronic and transport properties, the synthesis of non-hexagonal ring-hybridized carbon allotropes other than graphene presents considerable challenges. A bottom-up grown, ultra-flat biphenylene network is reported, comprising periodically arranged *sp*^2^ quaternary, hexagonal and octagonal units. The 4,4″-dibromo-2,2′,2″,5,5′,5″-hexafluoro-1,1′:4′,1″-terphenyl (DHTP) is firstly polymerized on the Au(111) surface via Ullmann coupling to form poly(2,5-difluoro-para-phenylene) (PFPP) as shown in Fig. 5D [39]. Subsequently, these chains undergo an on-surface interpolymer dehydrofluorination (HF-zipping) fusion for C−C coupling, resulting in a biphenylene network. On-surface chemical synthesis has significantly improved the success rate and accuracy of polymer synthesis while providing new insights into the synthesis of other non-benzenoid planar carbon allotropes.

## CHEMICAL REACTIONS IN SINGLE-MOLECULE JUNCTIONS

In 1959, American physicist Richard Feynman published a famous prophecy titled ‘There's Plenty of Room at the Bottom’, proposing a method that was completely different from the traditional top-down processing of materials and devices, which broadened the horizons of scientists. Twenty years later, in 1974, Aviram and Ratner from Northwestern University in the USA proposed the first theoretical model of a molecular rectifier, pointing out that a single molecule could unidirectionally export current along the molecular axis, predicting and demonstrating the potential feasibility of constructing single-molecule devices as electronic functional components, officially marking the birth of molecular electronics [40]. Subsequently, the SPM technology that rapidly developed in the 1980s provided initial technical support for studying the charge transport properties of molecules. In 1997, the mechanically controllable break junction (MCBJ) was born, which connected self-assembled single molecules to gold electrodes to measure their electrical properties [41]. Another method of preparing SMJs is to use carbon-based materials as the electrodes at both ends of the molecule. The selection of this electrode resulted in a significant difference in the testing of single molecules: the molecule is covalently bonded to graphene, and the break junction technology is no longer applicable. This brought a new testing method: *in-situ* real-time monitoring.

In general, SMJs, as an excellent platform, are applied for studying single-molecule chemical reactions, especially the reaction mechanisms and reaction dynamics [42]. Real-time and precise analysis of the intrinsic mechanisms of chemical reactions at the single-molecule level holds significant scientific research value. From a fundamental structural perspective, SMJs can be categorized into dynamic break junction techniques and static junction techniques [43]. In recent years, SMJs have witnessed significant advancements, achieving a balance between precision, controllability, reliability and scalability, thanks to their compatibility with various working conditions.

### Chemical reactions in dynamic single-molecule break junctions

The single-molecule break junction technique was proposed by Reed and Tour [44]. They utilized the MCBJ method to fabricate the first single-molecule device in this field. Subsequently, in 2003, Tao *et al.* proposed break junction technology based on STM [45]. The break junction technique utilizes the mechanical motion of nanotips or wires to gradually stretch or compress a molecule until the electrode-molecule-electrode junction breaks or forms, and the properties and behavior of individual molecules can be monitored by measuring the current, voltage or force of the molecular junction.

The SMJ technology provides a reliable method for studying single-molecule chemical reactions, with high-resolution electrical signal responses aiding in a deeper understanding and exploration of the rates, pathways and mechanisms of single-molecule chemical reactions. The Diels-Alder reaction is one of the most common reactions in chemistry. In typical chemical reactions, the orientation of molecules in gases or liquids is random and lacks directionality. Single-molecule electrical measurements based on STM break junction (STM-BJ) can reveal the average chemical coupling information of thousands of reactions. This allows researchers to control the dynamics of the approaching reactants. Coote's team discovered that for single-molecule structures of dienophiles with a fixed orientation, the reaction rate of the Diels-Alder reaction can be increased 5-fold when the polarity of the electric field promotes the flow of electrons from the dienophile molecule to the diene molecule (Fig. 6A) [46]. Experimental data indicate that when the bias was at −0.75 V, a total of 1116 product molecules were produced during the 6000 attempts to form molecular junctions in one hour, whereas only 252 product molecules were produced when the bias was at −0.05 V. This work represents the first confirmation of electrostatic catalysis in chemical reactions and quantitatively characterizes the impact of electrostatic forces on reaction rates using STM-BJ. To further substantiate the influence of the electric field direction on chemical reactions, the MCBJ technique and nuclear magnetic resonance (NMR) technology were employed to characterize the selective catalytic effect in a two-step cascade reaction involving Diels-Alder addition followed by aromatization, as shown in Fig. 6B [47]. The alignment of the oriented external electric field with the reaction axis of the SMJ enhances its catalytic efficiency by one order of magnitude in the aromatization reaction. Furthermore, the catalytic efficiency increases significantly with the increasing bias voltage, showing a strong field-dependence effect. However, the Diels-Alder reaction remains unchanged due to its reaction axis being orthogonal to the electric field. The selective catalytic effect of STM-BJ under an oriented external electric field reveals that single-molecule chemical reactions can be selectively manipulated through clever alignment between the electric field and the reaction axis.

Furthermore, to delve deeper into the mechanism of single-molecule electron catalysis reactions, Chen *et al.* utilized the STM-BJ technique, combined with ensemble electrochemical experiments, to construct a saturated 1,2-di(4-pyridinium)ethane (DPA^2+^) SMJ [48]. The experimental results indicate the transition from ethane to ethylene and reveal that positively charged molecular frameworks can serve as effective electron acceptors, receiving electrons injected from the electrode. This leads to redox reactions at the molecular junction, with the electric field promoting dehydrogenation, thus constructing a single-molecule redox switch. Single-molecule electrochemical tests combined with DFT calculations and ensemble electrochemical experiments elucidate the mechanism of the electron-catalyzed dehydrogenation reaction: electrons trigger the redox process and localized electric fields promote the dehydrogenation process.

For single-molecule catalytic reactions, catalysts can reduce the activation energy necessary for the transformation of reactants into products, subsequently changing the reaction rates. Activation energy, a fundamental element in the dynamics of chemical reactions, represents the energy required for molecules to transition from a stable state to a transition state. It is instrumental in predicting and comprehending both reaction mechanisms and rates. For instance, the level of activation energy has a direct influence on the chemical reaction rate. The faster a reaction occurs, the lower the exponent of activation energy it requires. Understanding activation energy allows us to determine reaction rates across varying temperatures and catalysts. Furthermore, the quantity and distribution of activation energy offer insights into the reaction's pathway. This implies that exploring more potent catalysts or modifying reaction conditions to reduce activation energy might be beneficial. Li *et al.* employed STM-BJ to real-time monitor the activation energy of the single Au–S bond breaking reaction during the catalytic process by halogen atoms [49]. By characterizing the maximum distance that the molecular junction can be stretched before the Au–S bond ruptures, known as the conductance plateau length, indirect information related to activation energy can be inferred. A shorter plateau length typically indicates a lower activation energy. For different halogen environments, it was found that the presence of halogen atoms, especially chlorine and iodine, can significantly reduce the breaking energy of the Au–S bond, suggesting that halogen atoms lower the activation energy. Combined with DFT calculations, the changes in the lowest value of the electron density (*ρ*_min_) along the axis between Au and S were analyzed. When halogen atoms are introduced, *ρ*_min_ decreases significantly, resulting in a corresponding reduction in activation energy and an easier rupture of the chemical bond. Therefore, this discovery provides quantitative physicochemical information for revealing the catalytic reaction mechanism with the analysis of activation energy.

Electric-field-induced molecular assembly is also a key issue in molecular devices. Among interactions that drive the formation of assemblies, the stacking interaction between π-conjugated molecular frameworks plays a crucial role in charge transport between molecules. Hong *et al.* found that with increasing electric field, the assembly probability of terphenyl molecules significantly increased, as shown in Fig. 6C [50]. Combined with DFT calculations, it is demonstrated that individual molecules tend to adopt a planar structure under strong electric fields, leading to an increase in the binding energy of the assemblies. By constructing model molecular systems at the single-molecule scale, this work elucidates the mechanism of electric-field-induced assembly of triphenylene molecules, providing a new strategy for using electric fields to regulate molecular assembly.

Additionally, keto-enol tautomerism is one of the fundamental chemical equilibria in organic chemistry. During the interconversion process between keto and enol forms, molecules can undergo significant π-electron rearrangements with relatively minor conformational changes, providing an ideal chemical basis for the design of single-molecule switches. However, there exists a contradiction between the high conversion stability and convertibility of the keto-enol tautomeric state. Hong *et al.* proposed a single-molecule reaction control strategy based on dual potential energy surfaces as shown in Fig. 6D [51]. Through bias control of charge injection into molecules, molecules can transition between neutral and cationic radical states, thereby achieving selective switching between the two potential energy surfaces. The keto and enol forms respectively dominate the equilibrium of tautomerism reactions under neutral and cationic radical potential energy surfaces, ensuring the stability of the tautomeric state. Both potential energy surfaces have unidirectional low tautomeric barriers, ensuring convertibility. Based on this theory, this work successfully fabricates a stable reversible single-molecule switch that can operate at room temperature, and discovers the significant role of electric fields in regulating chemical reaction processes.

In the study of molecular conformational transformations, photosensitive molecules have attracted widespread attention from researchers due to their controllability and high response rate. However, photosensitive molecules are currently mainly based on a relatively limited range of molecular units such as azobenzene, spiro compounds and diarylethene, which makes it difficult to meet the demands of photosensitive device development. Figure 6E demonstrates the implementation of the light-induced proton transfer (PIPT) strategy at the single-molecule scale for light modulation of non-photosensitive molecules. Specifically, the PIPT strategy controls the transfer of charges through single-molecule azulene junctions under ambient conditions using visible light, leading to reversible and controllable light-responsive molecular devices based on non-photoresponsive molecules and photoacids [52]. This approach builds highly reversible single-molecule light switches with switch ratios reaching an order of magnitude. Additionally, the application of PIPT in two single-molecule logic gates with electrical signal outputs, namely AND and OR gates, is demonstrated, providing new insights for the design and fabrication of novel photosensitive molecular devices.

In addition, SMJ technology has opened up new avenues for the study of complex biological enzyme molecules. Enzymes are a crucial class of protein machinery in living organisms that perform catalytic functions, providing reducing power and energy for cellular metabolic processes, and serving as receivers and transmitters of signals in the regulation of life activities [53–55]. While traditional ensemble-averaging approaches have been the primary means of studying enzyme catalysis, the precise characterization of enzyme catalysis can now be achieved using single-molecule characterization techniques. For instance, a representative catalytic cycle of formate dehydrogenase (FDH) has been successfully characterized by Hong *et al.* using single-molecule conductance experiments and multiscale simulations, revealing a reaction mechanism at the single-molecule scale that differs from the conventional textbook classic mechanism in the catalysis of formate dehydrogenase, as shown in Fig. 6F [56]. This study demonstrates that FDH exhibits different conductance values upon binding to the reduced or oxidized form of coenzyme I. This phenomenon can not only be used to distinguish between different reaction states in the catalytic cycle of FDH, but also serves as a marker for monitoring the trajectory of the catalytic cycle of FDH. This result differs from the classical Theorell-chance mechanism proposed by Nobel laureate Professor Theorell in 1955, as it does not involve a decoupling from the coenzyme state at the end of the catalytic reaction cycle. Combining multiscale simulations, this research proposes that, after the catalytic reaction concludes, the reduced form of coenzyme I bound in formate dehydrogenase directly converts to the oxidized form of coenzyme I through *in-situ* hydride ion transfer, subsequently initiating a new catalytic cycle. This novel mechanism is more energetically favorable. It is the first time that the dynamic process of enzyme catalysis has been observed at the single-molecule scale, and a new mechanism for formate dehydrogenase catalysis at the single-molecule scale has been proposed, providing a crucial foundation for subsequent research in single-molecule enzyme kinetics, enzyme design and engineering.

### Chemical reactions in static single-molecule junctions

Static SMJs as a stable electronic circuit, are more favorable for studying the intrinsic mechanisms of single-molecule chemical reactions. The higher stability and reliability of static junctions contribute to prolonged observation and analysis of single-molecule properties. Specifically, in static SMJs, chemical reactions in individual molecules can be induced and controlled by external fields such as light, electricity or magnetism. The kinetics, thermodynamics and electronic transport properties have been studied by monitoring the subtle changes in electrical signals. These studies contribute to a deeper understanding of chemical reactions at the molecular scale and have potential applications in the fields of molecular electronics and nanotechnology.

Graphene-based SMJs, represented as a static structure, are a notable approach. Guo *et al.* initially developed graphene nanogapped electrodes through dashed-line etching, followed by covalently bonding molecules to electrodes using amide linkages [57]. Graphene-based SMJs represent a prominent approach to static junctions. Subsequently, single molecules are covalently linked to the electrodes through amide bonds, resulting in a robust interface coupling between graphene and functional molecules. The stable covalent link between molecules and electrodes significantly enhances the device stability, ensuring the feasibility of prolonged detection of static molecular junction reactions in environments including solutions and low temperatures, and thereby driving the rapid advancement of SMJ research.

The static SMJ platform allows for *in-situ*, label-free and non-destructive sensing of molecular reaction processes at the single-event level, offering excellent time resolution. It serves as an excellent avenue for unveiling new mechanisms and phenomena that are obscured by ensemble averaging. In the research achievements obtained by Guo *et al.* in the field of graphene-based single-molecule devices, several single-molecule chemical reactions have been studied, including photoinduced isomerization reactions [58,59], nucleophilic substitution reactions [60], nucleophilic addition reactions [61], Fries rearrangement reactions [62], Diels-Alder reactions [63], Suzuki-Miyaura coupling reactions [64] and chiral stereochemical reactions. These observations not only validate the reliability of this platform, but also define important dynamic parameters such as rate constant, activation energy, energy difference of states and Gibbs free energy from real-time *I*–*t* data at the single-molecule level [9].

**Figure 7.**
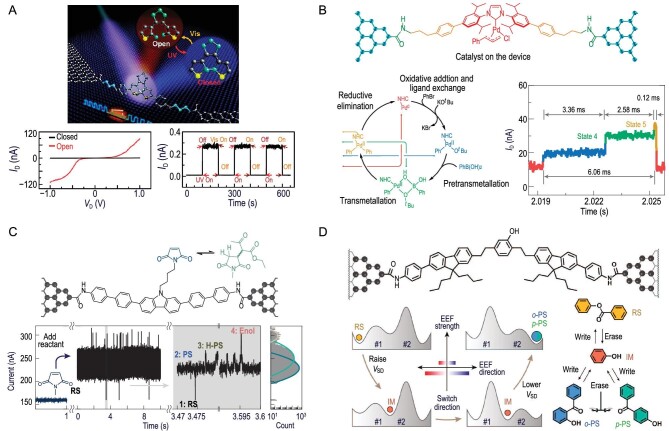
Single-molecule chemical reactions in graphene-based single-molecule junctions. (A) Single-molecule photoisomerization reaction of diarylethene molecule. Adapted with permission from ref. [58]. Copyright 2016 American Association for the Advancement of Science. (B) Visualization of Suzuki-Miyaura reaction. Adapted with permission from ref. [64]. Copyright 2021 Springer Nature Limited. (C) Chirality-induced spin selectivity for reaction. Adapted with permission from ref. [65]. Copyright 2023 Springer Nature Limited. (D) Single-molecule Fries rearrangement driven by an electric field. (RS, reactant state; IM, intermediate state; *o*-PS, ortho-product state; *p*-PS, para-product state). Adapted with permission from ref. [62]. Copyright 2022 Wiley-VCH GmbH.

In representative studies of photoinduced isomerization single-molecule chemical reactions, molecular engineering principles are applied to construct single-molecule static junctions using graphene electrodes with nanogaps and functionalization [58]. Through theoretical simulations, prediction and molecular engineering design, methylene groups are introduced between the diarylethylene functional center and the graphene electrode to reduce the coupling between the molecule and electrode. This successful optimization of the interface coupling resulted in a groundbreaking achievement in the stable and reversible monitoring of single-molecule photoinduced isomerization reactions (Fig. 7A).

Conducting catalytic reaction research using graphene-based single-molecule devices is crucial for precise manipulation and monitoring of reactions at the single-molecule level. The electrical signal response of the graphene-based single-molecule device confirms the mechanism of Pd-catalyzed Suzuki-Miyaura coupling, as shown in Fig. 7B [64]. Through the direct and precise monitoring of the Suzuki-Miyaura coupling reaction pathway by detecting sequential electrical signals generated by oxidation, addition/ligand exchange, pre-metallation, metallation and reductive elimination, it is demonstrated that the metal conversion is the rate-determining step in the catalytic cycle. This clarification resolves the disputed metal conversion mechanism and establishes the specific Suzuki-Miyaura coupling reaction pathway. Additionally, the kinetic and thermodynamic constants for each fundamental step and the overall catalytic timescale for Suzuki-Miyaura coupling are determined. This approach provides a feasible solution for real-time monitoring of transition-metal-catalyzed reactions.

Apart from the functional molecule, the electrode is also an important component in single-molecule devices, and effective control of molecules can be achieved through spin injection from the electrode. Due to the small differences between chiral molecule enantiomers, molecular conductance is often degenerate and difficult to distinguish. Graphene-based single-molecule devices offer a solution to distinguish chiral enantiomers by utilizing spin injection, as shown in Fig. 7C [65]. In the experiment, changes in chirality during the Michael addition process were directly monitored followed by proton transfer and keto-enol tautomerism within a single molecule. After the addition of ethyl acetate and alkaline solution, the *I−t* curve shows distinct current fluctuations corresponding to different reaction states, with four states clearly differentiated. The surface potential energy of the theoretically calculated Michael addition and its subsequent processes also exhibit a significant energy gap. Utilizing chirality-induced spin selectivity, the continuous current measurement of SMJs reveals chirality changes during the reaction.

High-sensitivity chirality identification provides a promising tool for studying symmetry-breaking reactions and unraveling the origin of chirality-induced spin selectivity. Furthermore, based on graphene-based single-molecule devices, the Fries rearrangement reaction not only allows for real-time monitoring of the reaction pathway at the single-molecule level, distinguishing interaction patterns between intra- and inter-molecular interactions, but also elucidates the Fries reaction mechanism [62]. This offers a powerful tool for future studies of molecular interactions (Fig. 7D). By controlling electrical input through the molecular junction circuit, it is possible to achieve repetitive switching between the stable RS and *p*-PS of the molecule. The initial stable state RS (*p*-PS or *o*-PS) can be converted into the intermediate state by applying a high voltage of +1 V (−1 V). Subsequently, it can be catalyzed by pyrophosphate to another stable *p*-PS (RS) state through the opposite −1 V (+1 V) electrical input. Utilizing the two binding sites in the phenol center (*ortho* or *para*) allows for multi-stable state transitions of the molecule to reconfigure the acyl moiety (resulting in different resistances). Based on the efficient switching of molecules between different structural states, a solid-state single-molecule memristor has been constructed, further achieving small physical dimensions, high performance and strong integration capability for single-molecule memristor devices.

## DETECTION OF CHEMICAL REACTIONS BASED ON NANOSTRUCTURES

The small size and high surface area of nano-structures enables extremely sensitive detection of low-concentration molecules, which is crucial for biomarker detection and drug development. Single-molecule detection leverages the unique properties of nanomaterials, such as nanopores, nanowires and nanotubes, to probe and analyze chemical reactions at the single-molecule level, thus detecting the chemical reaction dynamics and uncovering the reaction mechanisms. This technology holds vast potential for broad applications in biomedical research, materials science and chemistry.

### Single-molecule chemical reactions based on nanopores

The foundations of nanopore technology can be traced back to the 1950s when Coulter counting was used to calculate the number and size of suspended particles in electrolytes. Then, in the 1970s, came the introduction of single-channel current recording [66]. However, it was only in the late 1990s that *α*-hemolysin protein nanopores were proposed to investigate the translocation behaviors of single-stranded deoxyribonucleic acid (DNA) and single-stranded ribonucleic acid (RNA). This was the first time that the idea of using *α*-hemolysin for DNA sequencing was proposed, heralding a new approach in the research of nanopores for single-molecule detection [67]. Nowadays, nanopore technology has been developed as a real-time, highly sensitive approach for *in-situ* chemical and biological single-molecule detection [68–72].

The conventional nanopore detection mechanism is based on resistive pulse sensing. Two electrolyte-filled reservoirs are separated by a thin impermeable membrane and connected through a single nanopore [66]. Two electrodes are then added to the chamber, and a constant voltage is applied to the electrodes. The electrode with negative voltage is defined as ‘*cis*’, while the opposite side is defined as ‘*trans*’. The target molecules are driven through nanopores from ‘*cis*’ to ‘*trans*’ or in the opposite direction under the influence of applied electric fields and/or subsequent ion-selective electroosmotic flow within the nanopores. Due to the nanoscale inner diameter of the pore, the molecules passing through the pore can block the flow of ionic current, resulting in discrete current signals. After further analysis of the current signals, such as amplitude, noise and duration, one can obtain important information about the target molecules, including the charge, the shape and size, and the dipole moment.

Nanopore technology has been rapidly developing in various fields due to its advantages of ultra-sensitivity, low cost and being without labeling or modification. There are three different types of nanopores: biological [73–77], solid-state [78–80] and hybrid nanopores [81]. The biological nanopores are transmembrane protein channels assembled by pore-proteins on lipid bilayers, with uniform structures and highly reproducible dimensions. Solid-state nanopores are formed on materials, possessing good thermal stability, chemical stability and size controllability. Hybrid nanopores are the fusion of biological nanopores and solid-state nanopores, combining their common advantages and possessing excellent potential applications. These nanopores have developed the ability to decode molecular features with high throughput, providing important guidance for a comprehensive understanding of the properties and functions of single molecules.

**Figure 8.**
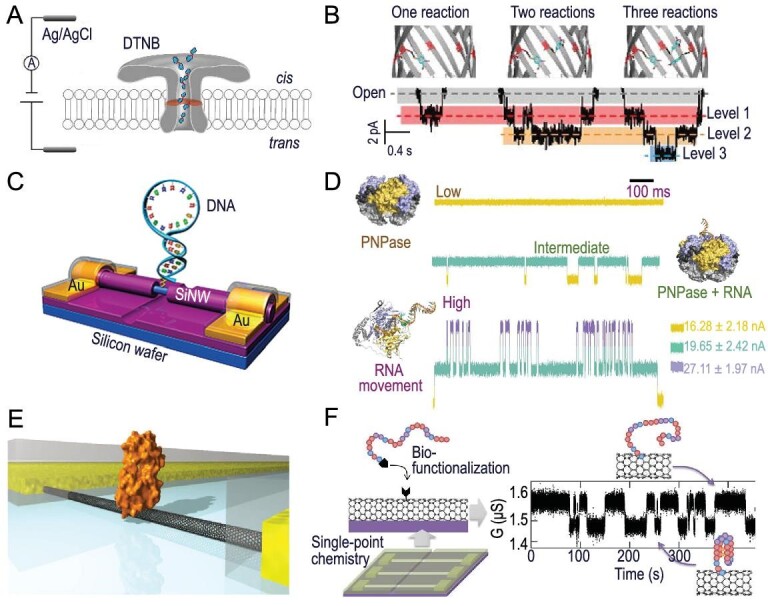
Single-molecule detection based on nanostructured devices. (A) Schematic diagram of detecting the DTNB reaction in the nanopore. (B) Reaction events in the nanopore at three levels. (A and B) Adapted with permission from ref. [84]. Copyright 2018 Science China Press and Springer-Verlag GmbH Germany. (C) Schematic diagram of a single-hairpin DNA-decorated SiNW biosensor. Adapted with permission from ref. [87]. Copyright 2016 Wiley-VCH Verlag GmbH & Co. KGaA. (D) Real-time electrical trajectories of the PNPase structural transformation based on SiNW. Adapted with permission from ref. [88]. Copyright 2023 Springer Nature Limited. (E) Schematic diagram of the single lysozyme being interrogated by a CNT-based device. Adapted with permission from ref. [89]. Copyright 2012 American Association for the Advancement of Science. (F) Single-molecule observation of DNA G-quadruplex folding in a CNT-based device. Adapted with permission from ref. [90]. Copyright 2016 American Chemical Society.

The most mature and widely used application of nanopore technology in single molecules is the accurate identification and analysis of molecular structure and properties, as well as the capture of the intermediate states and tracking of the dynamics. For instance, Bayley *et al.* utilized mutant protein nanopores to monitor the reactions of amino acid decomposition in real time and define the unstable intermediate products during the decomposition process [82]. The mutated protein was then extended to the study of the reaction mechanism between metal ions and the complexing agent *N*-propyliminodiacetic acid (PIDA), achieving the observation of complex covalent reaction processes at the single-molecule level [83]. In addition, Long *et al.* used a mutant aerolysin K238C to prepare the nanopores, and then replaced the 238-site lysine with cysteine, providing a reaction site for the binding of disulfide [84]. Subsequently, 5,5′-dithiobis-(2-nitrobenzoic acid) (denoted as DTNB) covalently bound to cysteine, and the disulfide bond formed between the DTNB molecule and the cysteine residue was determined through the blocking current (Fig. 8A and B). Furthermore, they revealed the formation and breaking events of individual chemical bonds in single-molecule chemical reactions at the sub-millisecond time scale based on the transient fluctuations of current in the nanopores, which guides the tracking of other complex chemical reactions through the mutant aerolysin.

To sum up, nanopores are suitable for investigating chemical reactions in an aqueous environment, with extremely high resolution, and are an effective way to explore reactions at the single-molecule level. Nowadays, there is still a need to develop new methods for placing the molecules completely and freely in any position in the nanopore channel, to achieve the construction of a nanopore reactor with a maximum degree of freedom for single molecules.

### Single-molecule detection based on 1D nanostructures

In 1991, Iijima *et al.* fabricated carbon nanotubes (CNTs), and the emergence of this 1D nanomaterial immediately garnered immense interest from scientists across various fields [85]. 1D nanomaterials, such as semiconductor nanowires and nanotubes, provide a novel approach for detecting single molecules due to their excellent electrical properties, fast signal response, large surface-to-volume ratio and size matching with single molecules. The sidewall of 1D nanomaterials can be modified at a single point to achieve single-molecule-level sensitivity. Among the 1D materials, silicon nanowire (SiNW)-based and CNT-based single-molecule biosensors can realize ultra-sensitive and label-free biological detection in a wide range of areas, such as clinical diagnostics, biomedical research and single-molecule sequencing. Here, the single-molecule detection based on SiNWs and CNTs will be introduced in detail.

SiNWs based on the field-effect principle are widely used in single-molecule detection with high sensitivity, especially in exploring the dynamics of random processes and molecular interactions in biological systems at the single-molecule level. This technique provides an excellent monitoring platform and endless opportunities for revealing genomic and proteomic information. Several ways to prepare SiNWs have been developed, including chemical vapor deposition, molecular beam epitaxy and an etching approach. The point connected with a single molecule on SiNWs can be precisely fabricated. Specifically, by using high-resolution electron beam lithography, a ∼5-nm-wide line is exposed at the specific position of SiNWs, and the window precursor is obtained. Then, wet etching is applied to completely remove the amorphous SiO_2_ layer in the window area. Through alkyne hydrosilylation of Si–H bonds with undecynic acid and *N*-hydroxysuccinimide (NHS)-esterification, active ester terminals for the subsequent attachment of single molecules are obtained. Single molecules with amino terminals can be covalently attached to single points on SiNWs. The changes in local charge density corresponding to points connected with a single molecule influence the conductivity of SiNWs. This method is called the point chemical modification method. By using this method, single biomolecules can be assembled into the SiNWs to construct a highly sensitive sensor, and then capture and interact with single molecules [86]. For instance, a single hairpin DNA molecule is captured by the side wall of SiNWs (Fig. 8C), and then temperature-dependent two-state oscillation signals of the current and the duration of the oscillation are obtained [87]. The oscillation is the conformational transition between the folded base pairing state and the unfolded random coiled state of hairpin DNA, with a time scale of several microseconds. This research not only strongly supports the ‘zipper model’ of DNA hybridization/de-hybridization, but also demonstrates the feasibility of real-time monitoring of internal hybridization and dissociation processes of a single DNA molecule.

In addition to identifying the conformations, SiNW-based devices can also be used to efficiently monitor the reaction process of biomolecules. In a recent study, Guo *et al*. present a reliable *in-situ* single-PNPase-molecule dynamic electrical detector based on a SiNW-based device with ultra-high temporal resolution. A single polynucleotide phosphorylase (PNPase) molecule is conjugated to the nanoscale special area on the surface of SiNWs (Fig. 8D) [88]. Real-time and label-free monitoring of RNA analog degradation including RNA analog binding, single-nucleotide hydrolysis and single-base movement, is realized at the single-base resolution. Statistical calculation confirms that the conformational entropy of PNPase drives the binding with RNA analogues. Based on these devices, a binding event of the enzyme (near the active site) with the nucleoside has been discovered, which offers a new approach to understanding the RNA degradation mechanism. By creating a fingerprint map for each site in the RNA analog sequence, it is possible to differentiate nucleotides at single-base resolution with an accuracy of ∼80% (for artificially designed sequences) and ∼79.17% (for the mccA gene sequence). Moreover, this single-molecule detection method based on single-point modified SiNWs can be widely applied to investigate the reaction mechanisms of enzymes and identify DNA, RNA and peptides.

Single-walled CNTs (SWCNTs) are also typical 1D nanomaterials composed of carbon atoms, and each carbon atom is connected by stable C−C covalent bonds. The excellent mechanical and electrical transport properties make them suitable for building stable devices. Experimentally, the approach of chemical vapor deposition is adopted to obtain high-quality and large-area CNTs. Since the diameter size of SWCNTs is perfectly compatible with organic molecules, SWCNTs can serve as a single-molecule electrical detector. Before the combination of single molecules, functionalized treatment is required on the surface of the SWCNTs. Point-functionalized SWCNTs connecting with single molecules have been widely reported in the study of biomolecules. For instance, a stable, high-bandwidth transducer for protein motion was produced by tethering a single lysozyme molecule to a CNT field-effect transistor [89]. Pyrene adheres to SWCNTs through π–π stacking, which provides diluted anchor points for a thiol to covalently conjugate with a single cysteine variant of T4 lysozyme (S90C) (Fig. 8E). The movement of the lysozyme molecule results in a change in the electrostatic potential, which is further quantified as a dynamically changing current, thus allowing electrical signal analysis to determine the lytic catalytic mechanism and activity of the lysozyme. In addition, single-point-functionalized SWCNTs have also been widely used in the study of conformational dynamics. A single-strand of DNA was covalently tethered to the single-point-functionalized SWCNTs and the process of reversible folding into a G-quadruplex structure was recorded in real time (Fig. 8F) [90]. The electrical measurements of the SWCNTs indicate that the electrical conductance of the molecule fluctuates between a high-conductance state and a low-conductance state, corresponding to the folded and unfolded forms of the G-quadruplex sequence. This is derived from the analysis of the characteristic lifetime of the high- and low-conductance states.

SiNWs and SWCNTs serve as two of the most popular 1D nanomaterials capable of detecting single-molecule dynamics, paving the way for the investigation of fundamental chemical mechanisms and reaction processes at the single-molecule level. Single-molecule point-functionalized SiNWs and SWCNTs have broad application value in the development of ultrasensitive and label-free biosensors.

## DETECTION OF CHEMICAL REACTIONS BASED ON SINGLE-MOLECULE FLUORESCENCE

Single-molecule fluorescence-based chemical reaction detection is a highly sensitive and selective analytical method that utilizes the fluorescent properties of molecules to detect and analyze single-molecule chemical reactions. In this method, molecules are typically labeled or modified to generate fluorescent signals, which are then detected to monitor the progress and outcomes of the reaction [13,14]. Single-molecule fluorescence detection allows for real-time monitoring of the reaction process, providing a profound understanding of molecular dynamics and dynamic processes.

### Single-molecule fluorescence detection

Single-molecule fluorescence measurement is a technique based on fluorescence emission, which can track the conformational changes, dynamics, interactions and manipulation of single molecules [13,91,92]. Due to the low background and high signal-to-noise ratio, it has become the most commonly used method for single-molecule detection [93–98]. The principle of fluorescence generation can be described by the energy level of the molecules. When a molecule absorbs photons or is excited by an electric field, the electrons will transition from the ground state to the excited state. Then, the electrons in the higher excited state return to the lowest vibrational level of the first excited state through vibrational relaxation and internal conversion without radiation. Finally, these electrons return to the ground state in the form of radiative transitions, emitting fluorescence. Using efficient single-photon counters to receive and count photons eventually enables the precise detection of single-molecule fluorescence [99].

The genesis of single-molecule fluorescence measurement dates back to 1976, when Hirschfeld *et al.* labeled a single antibody molecule with a luminescent group and subsequently observed fluorescent signals [100]. This marked the inception of the single-molecule fluorescence detection technology, enabling unprecedented insights into molecular behavior at the nanoscale. The 1980s witnessed significant advancements in this field. In 1982, a single lipid molecule using diffusible multiple fluorescent labeling techniques was successfully detected [101]. A truly remarkable breakthrough occurred in 1989, when Moerner and Kador, for the first time, observed the single-molecule limit in condensed matter through absorption spectroscopy, opening a new chapter in the development of single-molecule fluorescence measurement [102]. Since then, single-molecule measurement has gradually evolved from requiring low-temperature conditions to being feasible at room temperature, significantly expanding its practical applications. In 1995, Funatsu *et al.* employed total internal reflection microscopy to image a myosin molecule labeled with a single fluorescent group in an aqueous solution, subsequently tracking its flipping reaction [14]. With the continuous advancement of technology, the modalities of single-molecule fluorescence measurement have gradually diversified. Fundamental detection methods include photon burst detection, single-molecule image recording and single-molecule spectral mapping. The emergence of these techniques has enabled researchers to measure and record the fluorescent properties of single molecules with greater precision. As the 21st century dawned, the application expanded significantly, allowing for a broadened exploration of macromolecular interactions and dynamics. Concurrently, with the advancement of fluorescence resonance energy transfer (FRET) techniques, the integration of FRET with single-molecule fluorescence measurement has enabled the simultaneous provision of molecular information in terms of time, space and resolution [103]. Due to the high sensitivity of fluorescence signals and the minimal interference of photons on molecules, single-molecule fluorescence measurement is considered to be one of the most effective methods for real-time observation of single-molecule changes in organisms.

The key to single-molecule fluorescence detection is to ensure that only one molecule in the irradiated volume interacts with the laser. However, the optical diffraction limit always hinders single-molecule fluorescence detection. There are two commonly used methods to achieve this goal, near-field scanning optical microscopy [104–106] and far-field confocal microscopy [107–109]. For the first, the near-field scanning optical microscope can break through the diffraction limit. The basic imaging principle is to adjust the distance between the light source and the sample to a level close to nanometers, and the light source is confined by pinholes (or an optical system), so that the resolution no longer depends on the wavelength, but is determined by the size of the light source and the distance, thereby achieving a resolution of tens of nanometers. The near-field scanning optical microscope exhibits extremely high resolution and has been used to study phenomena such as single-molecule fluorescence imaging. However, there are drawbacks such as low output power, which leads to poor performance for sample detection. For the second one, the far-field confocal microscope uses a high numerical aperture objective lens to converge the laser beam into a diffraction-limited focal point and places a confocal hole with a diameter of 50–100 μm at the image plane to block the light outside the focal point. The fluorescence signal is received by the detector after passing through the confocal aperture, while non-focal plane light is filtered out by the confocal aperture. It has the characteristics of unrestricted excitation intensity, non-invasive detection and strong signal collection ability, which is very suitable for the study of single-molecule fluorescence.

### Fluorescence detection of small-molecule chemical reactions

Small molecules are important research objects in the field of chemical reactions. However, many mechanisms are still unexplored for small molecules, and for a deeper and more precise understanding of the structure–reaction relationship [4,92,110–115], it is necessary to further investigate various reactions *in situ* at the single-molecule level with sufficiently high temporal and spatial resolution.

One of the most critical aspects of understanding the nature of the chemical reactions of small molecules is to clarify the rate and mechanism of the reaction. For some small molecules with fluorescence characteristics, the fluorescence spectra can effectively reflect the information. For example, the 3′-(*p*-aminophenyl) fluorescein (APF) and resazurin fluorescent probes with high emission of fluorescence are used to reveal the activity of photocatalytic oxidation and reduction reactions located on a single bismuth oxybromide (BiOBr) nanoplate [116]. The resazurin is reduced by the photogenerated electrons in the conduction band of BiOBr and then produces the resorufin with a high fluorescence yield, while the photogenerated holes in the valence band oxidize APF to produce fluorescein. The rate of photocatalytic reactions can be precisely determined by collecting the fluorescence intensity at different sites on the BiOBr nanoplates.

**Figure 9.**
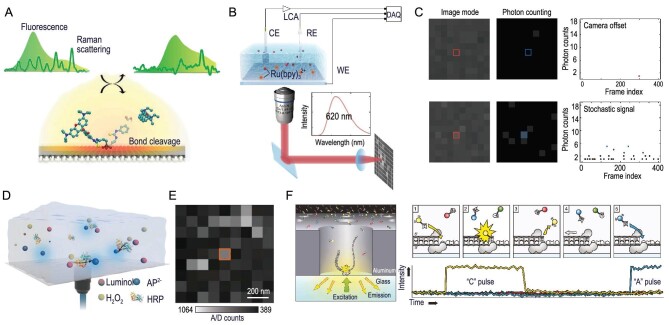
Fluorescence detection for single-molecule chemical reactions. (A) Detection of a photoinduced cleavage reaction of a rhodamine B isothiocyanate molecule by correlating simultaneous fluorescence and Raman spectroscopic signals. Adapted with permission from ref. [117]. Copyright 2020 American Association for the Advancement of Science. (B) The electrochemical recording set-up (upper panel) and widefield optical imaging (lower panel) for single-molecule electrochemiluminescence reactions. (C) The optical imaging of background signal (upper panel) and electrochemiluminescence signals (lower panel), as well as their respective photon counting results. (B and C) Adapted with permission from ref. [118]. Copyright 2021 Springer Nature Limited. (D) Schematic of chemiluminescence imaging system. (E) Imaging of single-molecule chemiluminescence signals. (D and E) Adapted with permission from ref. [119]. Copyright 2021 Springer Nature Limited. (F) Principle of single-molecule, real-time DNA sequencing based on ZMW. Adapted with permission from ref. [137]. Copyright 2009 American Association for the Advancement of Science.

In addition, combining fluorescence detection with other single-molecule spectroscopic methods can produce exceptional capabilities in terms of revealing chemical structures and reactions. Li *et al.* created a method of shell isolation of nanoparticle-plasmon substrates, which achieves a fluorescence enhancement factor of tens of thousands of orders and a Raman enhancement factor of millions of orders for joint measurement of fluorescence spectral and vibrational signals [117]. Through this method, *in-situ* spectroscopic information (Fig. 9A) on a series of reactions can be obtained, where a single rhodamine B isothiocyanate molecule undergoes the C−C bond cleavage and the phenyl carboxylic group removal under light irradiation, providing a novel and efficient monitoring technique for studying light–matter interactions and chemical reactions.

Revealing single-molecule chemical reactions from the perspective of single-photon imaging has always been a major scientific challenge, as the randomness of single-molecule chemical reaction processes and positions makes it difficult to effectively control and track. Recently, Feng *et al.* established an efficient electrochemiluminescence control system, which used spatially isolated molecular reaction localization information for optical reconstruction imaging [118]. It is the first wide-field imaging of electrochemiluminescence reactions beyond the optical diffraction limit (spatial resolution up to 24 nm) (Fig. 9B), making the direct imaging of single-photon-based single-molecule chemical reactions a reality. In this experiment, a single ruthenium-based molecule in the aqueous solution is triggered near the electrode surface and reacts with the free radicals generated by the electrode to emit photons. The diluted solution ensures that these molecules are spatially separated, while the extremely short lifespan of the free radical ensures that it only exists near the electrode surface at a very high dilution. Diluted molecules rarely encounter diluted free radicals, so each millisecond snapshot only captures one generated photon in one reaction, which can be confirmed by comparing the images of the background signal and electrochemiluminescence signals, as well as their respective photon counting results at an exposure time of 0.507 ms, as shown in Fig. 9C.

In addition, they further investigated the direct imaging of single-molecule catalytic luminescent reactions in solution (Fig. 9D) [119]. In the experiment, the reaction between luminol (5-amino-2,3-dihydro-1,4-phthalazinedione) and hydrogen peroxide, catalyzed by horseradish peroxidase, gave rise to the production of chemiluminescence. In order to visualize individual luminescent events, a single-molecule spatiotemporal isolation strategy is employed, which involves controlling the molecular density and isolating individual reactions in space and time. The focal plane was focused on the solution, and the photons generated by the reaction were collected through an optical lens and observed by an electron-multiplied charge-coupled device (EMCCD) camera, enabling single-photon detection with high signal-to-noise ratio (Fig. 9E). The imaging of fluorescence not only provides a profound understanding of chemiluminescent reactions but also serves as a powerful tool for analyzing activation energies and evaluating catalyst performance at the single-molecule level [120,121]. For instance, Li *et al.* conducted research on localized surface plasmon resonance-enhanced photocatalytic reactions based on single-molecule fluorescence detection. The reason for the enhanced catalytic performance is its ability to lower the activation energy of intermediate formation [121]. Specifically, the fluorescent oxidation reaction is catalyzed by plasmonic metal nanoparticles. This involved the conversion of non-fluorescent Amplex Red (AR) into the fluorescent product Resorufin (Rf) in the presence of hydrogen peroxide (H_2_O_2_). This reaction comprises two primary steps: initially, the AR is oxidized to form a non-fluorescent intermediate, AR•. Subsequently, this intermediate AR• is further converted into the final fluorescent product Rf. Among the two steps, the rate-limiting step is the initial oxidation of AR to form the non-fluorescent intermediate AR•. This process requires a relatively high activation energy, posing a bottleneck for the overall reaction rate. When plasmonic excitation is induced by irradiating gold nanorods with a 785 nm laser, the resulting hot charge carriers can effectively reduce the activation energy of the aforementioned rate-limiting step. Hot charge carriers facilitate the formation of OH•, a strong oxidant that can accelerate the oxidation process of AR, thereby reducing the energy required for the generation of the AR• intermediate. By measuring the fluorescent product, the reaction rate can be derived, and subsequently, the activation energy can be obtained from the slope of the reaction rate curve. Under plasmonic excitation, the activation energy for the intermediate formation step is reduced from 52.9 kJ/mol in the dark condition to 42.4 kJ/mol, significantly decreasing the energetic barrier of the reaction and thus accelerating the overall fluorescent oxidation reaction.

Furthermore, based on the nanoconfinement space provided by nanofluidic devices, which offer high spatial resolution comparable to the length scale of single molecules, it is possible to detect single-molecule chemical reactions in the solution. For instance, the manipulation and analysis of biological molecules (proteins, DNA) and chemical reactions (redox reactions and catalytic reactions) can be carried out by integrating nanofluidic devices with fluorescence spectroscopy and imaging techniques [122]. Nevertheless, fabricating nanoscale channel structures and manipulating individual molecules remain technically challenging, necessitating ongoing innovation and development to unravel the dynamics of chemical reactions at the single-molecule level.

Overall, the detection and analysis of single-molecule fluorescence is a refined approach to investigating the dynamics and interactions of small molecules, and it has great potential for future application in the visualization of chemical reaction sites.

### Fluorescence detection of single biomacromolecular reactions

Biomacromolecular reaction refers to the process in which organic compounds with large molecular weight, including proteins, nucleic acids and polysaccharides, undergo chemical or biological reactions within an organism. These reactions are the foundation of life activities such as metabolism, regulation and information transmission in organisms [123–127]. As the most representative macromolecular system, proteins play an important role in biological functions, as many physiological processes are related to structural changes in proteins [128–133]. Therefore, the intensive investigation of the structure of a protein is significant. Single-molecule fluorescence detection is a very mature technique to study the structures of proteins. Typically, fluorescent dyes are used to label proteins, enabling the observation of processes such as folding, conformational changes, aggregation and aggregation rates through fluorescence signals. This helps us gain a deeper understanding of the mechanisms underlying protein actions. For example, Forkey *et al.* used a single-molecule fluorescence polarization technique to measure the 3D structure of the light chain domain of brain myosin V [134], determined the orientation of single protein domains with 20–40 ms time resolution, and demonstrated that when the myosin molecule translocated along actin, a single fluorescent calmodulin light chain tilts back and forth between two angles. These results provide direct evidence for the lever arm rotation of the calmodulin-binding domain in myosin V. In addition to fluorescence labeling methods, single-molecule fluorescence resonance energy transfer (SM-FRET) is an important tool for investigating single biomacromolecules, which applies the scheme of distance-sensitive interactions between dipoles to measure the motion of molecules. In 1999, SM-FRET was first proposed to study the structural dynamics of a single Staphylococcal nuclease (SNase) molecule [117]. In the experiment, the fluorescent donor and fluorescent receptor are modified to two specific sites of SNase. Subsequently, the fluorescence resonance energy transfer efficiency of donor and receptor on a single SNase is observed and analyzed to reveal the fluctuations of different conformations between SNase molecules, which is attributed to the fluctuations in the protein backbone structure or side chains within the millisecond time scale. These results provide a new idea for the study of structure dynamics and folding of single protein molecules.

In addition, as one class of the most important protein molecules, enzymes can reduce the activation energy by interacting with substrates, changing the reaction path, and thus accelerating the rate of chemical reactions, which has always been a focus of research. A commonly used approach for the study of a single biological enzyme is fluorescent labeling [128], and according to the fluorescence intensity change of the substrate before and after the enzyme-catalyzed reaction, the reaction rates and mechanism of the enzyme can be obtained. For instance, Krischner *et al.* investigated the process by which the proteasome catalyzes the substrate [135]. By fixing the proteasome on the surface of the glass substrate, different conformations of ubiquitin chains connected to the substrate were constructed, and each ubiquitin was labeled with fluorescent molecules. The degradation of the substrate by the proteasome catalysis is monitored by observing the fluorescent dots. When the substrate is completely degraded, the fluorescent dots disappear. The results showed that the degree of substrate ubiquitination influences the interaction of the proteasomes with the substrates, and the structure of the ubiquitin can impact the process of the substrate entering the proteasome channel, eventually affecting the degradation of the substrate. A significant example of the enormous potential of single-molecule spectroscopy in studying complex enzyme molecules is the random biochemical reactions of individual enzyme molecules observed in real-time by Xie *et al.* using far-field fluorescence imaging [136]. They chose the flavonoid adenine dinucleotide (FAD) contained in the active site of cholesterol oxidase, which can reversibly switch between fluorescent and non-fluorescent forms during the catalytic process. According to the trajectory of fluorescence emission intensity, it was observed that each fluorescence ‘on/off’ corresponds to a cycle of enzyme molecule catalytic state, achieving real-time observation of enzyme oxidation and reduction states in a single catalytic reaction. In addition, this study also revealed that the catalytic rate constant of a single enzyme molecule is not fixed but fluctuates over time, that is, the waiting time (duration) of each oxidation and reduction state is random and has a strong memory effect. This opens up a new field for single-molecule spectroscopy to study the specific mechanisms of enzyme interactions. At present, most of the enzymatic reaction experiments are carried out outside of living organisms. However, in a more complex intracellular environment, how to ensure that single-molecule fluorescence is not quenched, how to track the variation of fluorescence in real-time, and how to attach specific enzymes to the fluorescent probes are urgent problems to be solved in the future. The vast majority of biochemical reactions are based on the manipulation of enzymes, so the investigation of the single enzyme molecule plays a crucial role in better understanding life activities for humans, helping us to find the most favorable reaction conditions to maximize the efficiency of enzyme-catalyzed reactions.

Fluorescence detection of single biological macromolecule chemical reactions has important applications in precision sequencing and diagnostics. For example, single-molecule sequencing can be realized using a DNA polymerase anchored to the bottom of a zero-mode waveguide (ZMW) nanostructure array (Fig. 9F) [137]. The DNA polymerase is synthesized by four distinguishable fluorescently labeled deoxyribonucleoside triphosphates (dNTPs). The DNA sequence is determined by detecting the fluorescence of correctly base-paired phospholinked dNTPs binding to the active site of the polymerase. Specifically, the principle of single-molecule, real-time DNA sequencing based on ZMW nanostructure can be described as follows: (i) a phosphorylated nucleotide binds to the template homologously at the polymerase active site; (ii) an increase in fluorescence output in the corresponding color channel is then detected; (iii) formation of phosphodiester bonds releases dye-linker-phosphate products, which diffuse out of the ZMW, thereby ending the fluorescence pulse; (iv) the polymerase translocates to the next position; and (v) the next homologous nucleotide binds to the active site, starting the next pulse. These ZMW nanostructure arrays can detect thousands of single-molecule sequencing reactions simultaneously. DNA synthesis over thousands of bases without steric hindrance can be observed based on conjugating fluorophores to the terminal phosphate moiety of the dNTPs.

## DETECTION OF CHEMICAL REACTIONS BASED ON CROSSED MOLECULAR BEAMS

The CMB technique is an effective experimental approach for the in-depth investigation of intermolecular interactions and reaction dynamics. Based on the collision of crossed molecular beams, this technique allows for the observation of dynamic behaviors of molecules during collisions under precisely controlled experimental conditions, thereby unveiling the fundamental nature of reaction kinetics. The principle of the CMB technique lies in the formation of a cross-collision between molecular beams of different origins in a high-vacuum reaction chamber, resulting in single collisions and scattering among molecules. Multiple windows placed around the scattering chamber allow for the detection of information such as the energy distribution, angular distribution of ions and electrons, and molecular energy states of product molecules as well as elastically scattered reactant molecules [138–140]. Owing to constraints in the accuracy and resolution of experimental apparatus, initial advancements with regard to the CMB technique progressed at a relatively unexceptional pace. Nevertheless, during the 1980s, noteworthy breakthroughs in the realm of laser technology, mass spectrometry and computational chemistry approaches resulted in a marked enhancement in the resolving capacity and sensitivity of CMB experiments. During this period, scientists such as Herschbach and Lee profoundly enhanced and advanced the technique, solidifying its position as a formidable instrument for investigating molecular collision dynamics [141,142]. Subsequently, in 1986, Herschbach and Lee were honored with the Nobel Prize in Chemistry, in recognition of their exceptional contributions to the field of CMB research. In the 21st century, the CMB technique has entered a stage of high-quality development [143–145]. Yang *et al.* developed a new generation of high-resolution and high-sensitivity scientific instruments for CMB experiments, achieving significant breakthroughs in the field of elementary chemical reaction dynamics research. Through a combination of precise scattering experiments and theoretical research, they have unveiled various resonant phenomena in chemical reactions, propelling the study of chemical reaction transition states at the quantum level [146]. For instance, the highly sensitive H atom Rydberg tagging time-of-flight method was used by Yang *et al.* to conduct a CMB scattering study of the F + H_2_ → HF + H reaction with full quantum-state resolution. As a result, pronounced forward-scattered HF products were observed at a collision energy of 0.52 kcal/mol, which is due to both the ground and the first excited Feshbach resonances trapped in the particular vibrationally adiabatic potential, with great enhancement by interference between the two resonances. This research achieves the definitive detection of reaction resonances in the F + H_2_ → HF + H reaction in a scattering experiment.

**Figure 10.**
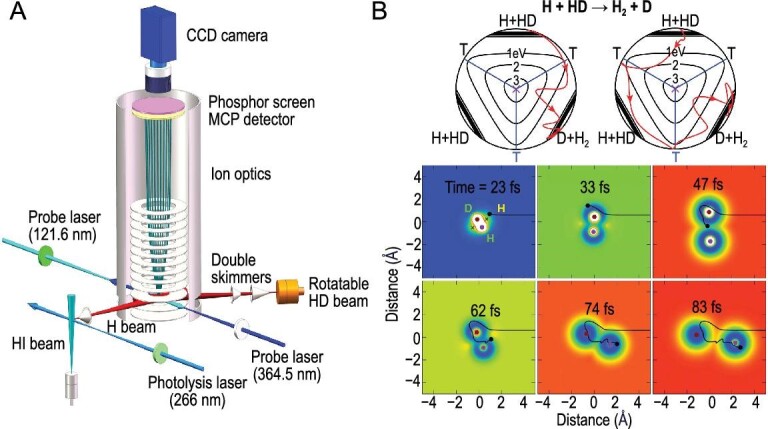
The research of chemical reaction dynamics based on crossed molecular beams. (A) Schematic of the experimental set-up. Adapted with permission from ref. [147]. Copyright 2018 Macmillan Publishers Limited, part of Springer Nature. (B) Representative direct and roaming trajectories in hyperspherical coordinates for the H + HD → H_2_ + D reaction. Adapted with permission from ref. [148]. Copyright 2020 American Association for the Advancement of Science.

The CMB technique also plays a crucial role in observing fine quantum-mechanical structures in product angular distributions that are important to fundamental studies of chemical reaction dynamics. As the simplest chemical reaction in nature, the H + H_2_ reaction serves as the benchmark system for studying chemical reaction dynamics. Quantum dynamics theory predicts the forward angular oscillation in the H + H_2_ reaction. Researchers have confirmed this theoretical prediction by observing the fast forward-scattering oscillations in the product angular distribution of the H + HD → H_2_ + D reaction in CMB experiments [147]. The H-atomic beam is generated by the photodissociation of HI and collides with the HD molecular beam at a crossing angle of 150°. The D atom produced in the collision zone was detected by a two-color (vacuum ultraviolet (VUV) + ultraviolet (UV)) threshold ionization method (Fig. 10A). At a collision energy of 1.35 eV, obvious oscillatory structures are observed for the H_2_ (*v*′ = 0, *j*′ =1, 3) product state, which is in great agreement with quantum-mechanical dynamics calculations. The analysis of the results reveals that the oscillatory forward-scattering components are mainly contributed by the total angular momentum around *J* = 28. The partial waves and impact parameters responsible for the forward scatterings are also determined from these observed oscillations, providing crucial dynamics information on the transient reaction process. This research will contribute to unraveling more quantum properties in chemical reactions.

In-depth research based on the CMB technique has continuously revealed quantum effects in chemical reactions. Yang's team discovered a new quantum geometric phase effect in the simple chemical reaction of hydrogen atoms with hydrogen molecules (H + HD → H_2_ + D) based on the CMB technique (Fig. 10B) [148]. Quantum interference phenomena are common in chemical reactions, but understanding the roots of these interference phenomena accurately is challenging. This study focused on the simplest reaction system involving H + H_2_ and its isotopes. One reaction pathway represents a direct process, where H collides and directly removes the H atom from HD. The other reaction pathway corresponds to a roaming mechanism, where H collides and ‘roams’ within HD for a while before removing the H atom. The hydrogen molecules produced by these two different types of reaction pathways converge at specific scattering angles, leading to systematic oscillations in the reaction product, hydrogen molecules. Interestingly, within the explored collision energy range, reactions occurring through the roaming mechanism account for only ∼0.3% of the overall reactivity, but can be elucidated through both theoretical and experimental means. This study creatively developed a new method based on topological principles to analyze the pathways of chemical reactions.

Studying the dynamics of chemical reactions using the CMB technique is beneficial for a deeper understanding of chemical processes with atoms as the subject of reactions, revealing the roles and behaviors of molecules in chemical reactions, and aiding in further comprehension of quantum effects.

## OUTLOOK

In summary, this review provides a comprehensive overview of single-molecule chemical reaction dynamics based on various technological platforms such as SPM, SMJ, single-molecule nanostructures, single-molecule fluorescence detection and CMB. It summarizes the advantages, limitations and future directions of several techniques for studying single-molecule reactions, as shown in Table 1. Single-molecule chemical reaction dynamics aims to reveal the behavior of individual molecules, and discover chemical reaction processes and mechanisms that are difficult to elucidate by ensemble averaging experiments. Simultaneously, it supplements and advances existing chemical reaction theories, potentially addressing critical scientific issues in the fields of physics, chemistry, biology, materials and beyond. It represents one of the current frontiers in scientific research. Single-molecule chemical reaction dynamics is an emerging and continually evolving frontier discipline built upon the foundation of single-molecule science. Its development benefits from advancements in nanofabrication and multidimensional characterization technologies. However, it is still in its early stages. To further advance single-molecule chemical reaction dynamics, several challenges need to be overcome.

**Table 1.** Several techniques for investigating single-molecule reactions.

Techniques	Advantages	Limitations	Developability	Refs
**STM** ^a^	Atomic precision; no sample damage; time scale: ns	High surface flatness and ultra-fine probe; not applicable to insulators; usually small molecules	Ultra-fine and ultra-sharp probes; enhance temporal and spatial resolution; integration of STM with other modules	[5,6,15]
**AFM** ^b^	Atomic precision; wide range: conductor, semiconductor, insulator; timescale: ns	Sample damage; ultra-high vacuum for qPlus-AFM; poor chemical resolution; usually small molecules	Ultra-fine and ultra-sharp probes; enhance temporal and spatial resolution; integration of AFM with other modules	[5,7,19]
**Dynamic SMJ** ^c^ **: STM-BJ** ^d^ **, MCBJ** ^e^	Convenient and effective operation; high repeatability and good statistics; time scale: μs	Single means of regulation; low test accuracy; confined to the solution state	Improving device accuracy; expanding the range of test samples; new means of regulation	[9–11]
**Static SMJ: Graphene-SMJ**	Stable molecular electrode covalent connection; stable device performance; high test accuracy; diversity of regulatory means; timescale: ns	Long device preparation cycle; limitations of molecular anchoring groups	Optimize the preparation method; ultra-cold detection, ultra-fast detection, and visualization; integration of functional devices	[9,57,58]
**Nanopores**	No marking or embellishment requirement; ultra-high sensitivity; timescale: sub-μs	Confined to the solution state; high interference from vibration noise and heat sources	Deepen the application in the biological field; optimize DNA, RNA and protein sequencing technologies	[66,73]
**1D nanostructures** ^f^	No marking or embellishment required; high test accuracy; timescale: ns	Uneven sizes and irregular morphologies; complex preparation process	Optimize protein sequencing and virus detection technologies; development of label-free biosensors	[86,89]
**SM fluorescence** ^g^	High sensitivity and selectivity; low background and high signal-to-noise ratio; timescale: μs	Need marking; sample damage after labeling or modification	Development of medical diagnosis and analysis technologies; ultra-sensitive methods for monitoring environmental pollutants	[1,14,92]
**BMB** ^h^	Strong flexibility and tunability for the energy and angle; highly accurate and detailed information; timescale: fs	Processing speed and sample characterization area; sophisticated equipment	Optimized equipment; development of new molecular beam sources; integration of CMB with other modules	[138–140,144]

a Scanning tunneling microscope. ^b^Atomic force microscope. ^c^Single-molecule junction. ^d^Scanning tunneling microscope break junction. ^e^Mechanically controllable break junction. ^f^1D nanomaterials. ^g^Single-molecule fluorescence. ^h^Crossed molecular beam.

### Development of single-molecule characterization techniques

The focus is on developing ultra-high spatial and temporal resolution characterization techniques, integrating various physical property measurements, such as electrical, optical, mechanical, magnetic and thermal properties. This involves advanced detection methods like ultra-cold, ultra-fast and visualization techniques. The goal is to establish precise multidimensional and multimodal methods for characterizing single-molecule systems. This will enable accurate characterization of the processes related to material transformation, energy transfer and electron transfer, providing high-resolution characterization and control of weak interactions in complex systems. Furthermore, ultrafast spectroscopic techniques can be used to achieve real-time, *in-situ* spatial characterization of single-molecule dynamics, thereby expanding our understanding of complex chemical reactions. The development of these advanced characterization techniques holds the promise of further exploring the fundamental principles of chemical reactions.

Single-molecule experiments provide strong technical support in revealing the essential mechanism of chemical reactions, but for extreme conditions, there are still many new phenomena in dynamics and chemical reactions that need to be explored. Ultra-cold molecular physics has always been regarded as an entrance to the field of quantum science [149–153], and its development can be traced back to the 1980s when researchers cooled atoms and then observed the inherent quantum behavior of atoms through the laser cooling technique. However, the energy level of the molecule is more complex, and the vibrational and rotational energy levels cannot undergo cyclic transitions, making it extremely difficult to cool the molecule to a temperature similar to that of the atom. The emergence of various new technologies has rapidly increased investigation into ultra-cold molecules, in which magneto-optical trap technology can cool the molecules to extremely low temperatures, and some basic research topics have been developed [154,155]. Observing single-molecule chemical reactions in ultra-cold environments, where molecular kinetic energy is dramatically reduced, allows for the observation and exploration of microscale interactions between molecules, thus aiding in the elucidation of the quantum mechanisms involved in chemical reactions. It is believed that with this advancement in research, the door to comprehensive coherent manipulation of all degrees of freedom of molecular quantum systems will be opened.

Ultra-fast techniques based on femtosecond laser pulses play an important role in revealing the dynamics of chemical reactions, as the chemical reactions typically occur on the femtosecond timescale [156–158]. In 1999, Zewail *et al.* used an ultra-short laser pulse to stimulate molecular beams, revealing the molecular dynamics on a femtosecond timescale, and then accurately manipulated the yield of chemical reactions through the interaction of laser pulses and molecules, which resulted in revolutionary changes in the theory and experiments of chemical science [159]. Investigation based on the interaction of single molecules and ultra-fast light sources has also developed rapidly in recent years [160–163], which greatly promotes the development of ultra-fast coherent control technology for the dynamics of single-molecule chemical reactions. Nowadays, the field of ultra-fast science has entered the attosecond world. It is believed that the application of attosecond laser technology to the single molecule field can explore electronic dynamics during chemical reactions.

The dynamics of single-molecule chemical reactions play a crucial role in both basic science and practical applications. Due to its fast rate, complex mechanisms and the inherent randomness of processes and positions involved, tracking and controlling chemical reactions at the single-molecule level can be highly challenging. Therefore, visualization of single-molecule chemical reactions has always been the goal. Fortunately, research into the visualization of the chemical reactions of single molecules has gradually developed in recent years. For instance, imaging of a single photon produced by chemiluminescence reactions in a solution has been achieved. Based on the platform of single-molecule devices, the rapid probing of the changes of conductance in the catalytic functional center of a single molecule has achieved visualization of the structural changes and reaction paths of the catalyst in chemical reactions [65]. Therefore, the use of SPM techniques holds promise for further enabling the visualization of single-molecule chemical reactions in *in-situ* environments, and 3D imaging through transmission electron microscopy (TEM) technology can facilitate molecular reaction imaging. The development of visualization techniques offers limitless opportunities for research in the fields of chemistry and biology.

### Exploration of the intrinsic processes of single-molecule chemical reactions

Single-molecule chemistry focuses on tracking chemical reactions at the single-molecule level, particularly reaction kinetics, which cannot be replaced by other characterization methods. With developed single-molecule multidimensional characterization techniques, the reaction pathways of single molecules should be precisely mapped. This eliminates the ambiguity introduced by averaging effects and allows the capture of reaction intermediates and potential transition states produced during the reaction. Analyzing the reaction kinetics at the single-molecule level offers an effective means to explore the true pathways and principles of molecular reactions.

### Advancement of theories for single-molecule chemical reactions

Single-molecule science is an ideal platform for studying non-equilibrium statistics and quantum effects in individual quantum systems. For single-molecule chemical reactions, classical dynamics theories and models are no longer applicable. The establishment and refinement of new dynamical theoretical models and mechanisms from a quantum and atomic perspective are required to simulate the entire process of single-molecule chemical reactions. Combining previous fragmentary theoretical models with the latest artificial intelligence (AI) technologies, such as deep learning, and utilizing quantum computing allows for the theoretical simulation and modeling of the entire process of single-molecule chemical reactions. Therefore, there is an urgent need to establish theoretical models that are more practical, develop simulation methods across multiple time and spatial scales, and advance quantum simulation methods based on quantum computing. The development of these theoretical approaches provides crucial scientific tools for in-depth investigations of single-molecule chemical reaction processes. Simultaneously, they build upon precise theoretical characterizations of single-molecule systems, leverage the pioneering advantages of theoretical calculations, propose new mechanisms, concepts or designs, and provide theoretical insights and pathways to guide and drive related experimental research. In addition, due to the failure of single-molecule systems to meet statistical conditions, it is necessary to redefine concepts such as temperature derived from the energy's Boltzmann distribution, as well as fundamental thermodynamic quantities like entropy and pressure in macroscopic systems. This may present new opportunities for the study of single-molecule theories and mechanisms.

### Breakthroughs in emerging interdisciplinary fields of single-molecule sciences

The development of single-molecule chemical reaction dynamics allows researchers to gain a deeper understanding of the structure and properties of individual molecules, explore details of molecular behaviors, and acquire information that macroscopic analytical methods cannot provide. Moreover, single-molecule science is a highly interdisciplinary and open frontier research field. The incorporation of tools like AI and machine learning will enhance the foresight and interdisciplinarity of single-molecule chemical reaction research. In the future, deep integration with physics, chemistry, biology and informatics is expected to create new areas of growth and technological breakthroughs. This has the potential to unveil the intrinsic mechanisms of material transformations and life phenomena, thereby addressing numerous fundamental scientific challenges in traditional disciplines and ensemble average experiments. The study of single-molecule chemical reactions provides strong support for the design of novel functional molecules and catalysts. By gaining in-depth insight into molecular-level reaction mechanisms, it becomes possible to precisely design new molecules and catalysts with specific properties and activities. Furthermore, achieving quantum bits through precisely controlling single molecules, and using the specific properties or states of molecules, such as molecular spin or electronic excited states as carriers of quantum information, holds potential for the development of a new generation of quantum information technology. This capability is expected to pave the way for innovative advancements in the field, offering a highly controlled and compact platform for quantum information processing with significant potential for applications in the fields of quantum computing and quantum communication. Lastly, it promotes the in-depth cross-disciplinary integration of single-molecule science with medicine and biology to address critical issues in life sciences, with a focus on improving human health and generating revolutionary applications.
